# The gut microbiota remodes amino acid and lipid metabolism in incomplete revascularization of CHD with phlegm-dampness syndrome: an integrated multiomics and network pharmacology study

**DOI:** 10.3389/fmolb.2026.1748007

**Published:** 2026-03-09

**Authors:** Xinyu Zhang, Wei Jiang, Xiaoqing Li, Wenjing Xu, Tong Liu, Haiming Cao, Haining Zhao, Wenhua Shi, Taohua Lan, Weihui Lu

**Affiliations:** 1 The Second Clinical Medical College of Guangzhou University of Chinese Medicine, Guangzhou, China; 2 State Key Laboratory of Dampness Syndrome of Chinese Medicine, The Second Affiliated Hospital of Guangzhou University of Chinese Medicine, Guangzhou, China; 3 Department of Cardiology, Guangdong Provincial Hospital of Chinese Medicine, Guangzhou, China; 4 Chinese Medicine Guangdong Laboratory, Hengqin, Guangdong, China; 5 State Key Laboratory of Traditional Chinese Medicine Syndrome, Guangdong Provincial Hospital of Chinese Medicine, Guangzhou University of Chinese Medicine, Guangzhou, China

**Keywords:** coronary heart disease, gut microbiota, incomplete revascularization, network pharmacology, phlegm-damp, untargeted metabolomics

## Abstract

**Background:**

Incomplete revascularization of coronary heart disease (IR-CHD) is a novel category of CHD that has developed; such patients often have persistent angina of unknown etiology, which seriously affects quality of life and prognosis and urgently needs in-depth study.

**Methods:**

Taking IR-CHD patients with PD Syndrome, non-PD (NPD) Syndrome, and healthy individuals as research subjects, through the integration of microbiomics and metabolomics studies of clinical samples and the network pharmacology research strategy of three classic TCM formulae, we systematically explored the biological basis of TCM Syndrome differentiation for PD Syndromes of IR-CHD.

**Results:**

IR-CHD patients with PD Syndrome demonstrated a unique metabolic profile and gut microbiota structure characterized by an increase in branched-chain amino acid metabolism and a decrease in glycerophospholipid metabolism, and 6 Syndrome-specific differential metabolites (DMs) were identified. Additionally, combined analysis of the gut microbiota and metabolites revealed that differential gut microbiota (DGMs), including *Muribaculum*, *Odoribacter*, and *Agathobacter*, may be involved in metabolic disorders associated with amino acids and lipids in PD syndrome. *Agathobacter*, *Odoribacter* and 3-methyl-2-oxo-pentanoic acid might be potential biomarkers for PD Syndrome by ROC diagnostic analysis. Furthermore, a comprehensive network pharmacology and multiomics analysis suggested that PD Syndrome formulae regulation overlaps with the metabolic disorder pathway mediated by the gut microbiota in PD syndromes, that is, the PD Syndrome formula may regulate the intestinal microenvironment and improve metabolic disorders through the PD Syndrome-specific pathway in IR-CHD patients.

**Conclusion:**

IR-CHD patients with PD TCM Syndrome have amino acid and lipid metabolic disorders and that the gut microbiota plays an important role in their metabolic regulation. This study also provides an evidence-based strategy for exploring the biological basis of TCM Syndrome differentiation, which is helpful for the translation of TCM theory into precision medicine practice.

## 1 Introduction

Coronary heart disease (CHD) is the leading cause of morbidity and mortality worldwide (China and Hu, 2023). Coronary revascularization, including percutaneous coronary intervention (PCI) and coronary artery bypass graft (CABG) surgeries, serves as the cornerstone therapy for severe stenosis or occlusion, aiming to alleviate angina, enhance quality of life, and reduce cardiovascular event risks (Lawton et al., 2022). According to the China Cardiovascular Intervention Forum (CCIF) report, 919,256 patients underwent coronary interventional therapy for CHD in China in 2018, establishing postinterventional CHD as a distinct clinical entity (Han, 2012, Cardiovascular Disease Committee of the World Federation of Chinese Medicine Societies et al., 2020). While the quality and volume of coronary interventions demonstrate steady progression, coronary revascularization itself may induce coronary and myocardial injury. Notably, 20%–34% of revascularized patients experience recurrent angina under standard pharmacotherapy despite resolved macrovascular stenosis, significantly impairing quality of life and prognosis (Cardiovascular Disease Committee of the World Federation of Chinese Medicine Societies et al., 2020; Alexander et al., 2008). This phenomenon is correlated with thrombogenesis, coronary spasm, stent restenosis, untreated stenosis, microvascular dysfunction, and other psychological factors (Abbate et al., 2007).

Although repeat revascularization may address post-PCI restenosis in select cases, substantial patient populations decline reintervention. Moreover, contemporary medicine lacks targeted therapies for patients with prior CABG, multivessel PCI, coronary microvascular dysfunction, or diffuse graft vasculopathy (Abbate et al., 2007). Therefore, elucidating the pathological mechanisms and biological basis underlying incomplete revascularization (IR) and advancing etiological characterization will inform the development of targeted therapeutic strategies.

As a medical system practiced in China for millennia, Traditional Chinese Medicine (TCM) centers on the principle of “syndrome differentiation and treatment” (Bian Zheng Lun Zhi). This foundational methodology prioritizes dynamic disease diagnosis and personalized therapeutic strategies based on the identification of specific Syndrome (ZHENG) patterns (Su et al., 2014). According to TCM, the main pathogenesis of CHD is heart meridian obstruction (Xin Mai Bi Zu), which is a syndrome characterized by differences in origin and excess superficiality (Ben Xu Biao Shi). This obstruction arises from pathological factors such as Qi stagnation, blood stasis, cold congelation, and phlegm damp, which can block the heart meridian. According to TCM principles, “obstruction leads to pain” (Bu Tong Ze Tong). Coronary revascularization, as an exogenous trauma, shares pathophysiological parallels the TCM concept of “heart meridian obstruction”. An epidemiological survey of 976 CHD patients revealed that phlegm-damp (PD) syndrome was used as a single syndrome element, accounting for 71 patients. 41% (Mei and Zeng, 2019). Post-PCI syndrome profiling revealed that PD is the predominant pattern. Moreover, patients with PD syndrome have more severe coronary artery lesions and a greater risk of poor prognosis (Sun, 2011). Against the background of modern medicine, PD TCM Syndrome is closely linked to the pathophysiology of CHD, including lipid metabolism disorders, inflammatory responses, insulin resistance, and hemorheological abnormalities (Huang et al., 2018; Wang et al., 2013; Huang et al., 2024).

The accumulation of high-quality evidence from TCM interventions in postcoronary revascularization studies confirms their potential to identify critical intervention targets, offering new perspectives on incomplete revascularization in CHD (IR-CHD) patients. However, elucidating the scientific foundations of TCM syndrome differentiation remains challenging owing to the paucity of objective and standardized metrics for syndrome classification and therapeutic evaluation. Systems biology, as a research methodology that integrates multilevel biological information such as metabolomics, microbiomics, and transcriptomics, is highly consistent with the complex scientific theory and thought processes underlying the concept of syndrome in TCM (Hood, 2018). Gaosong Wu et al. employed an integrative systems biology approach, incorporating proteomics, metabolomics, and network pharmacology, to systematically investigate the biological basis of two Syndrome patterns in CHD (Wu et al., 2021).

**FIGURE 1:**
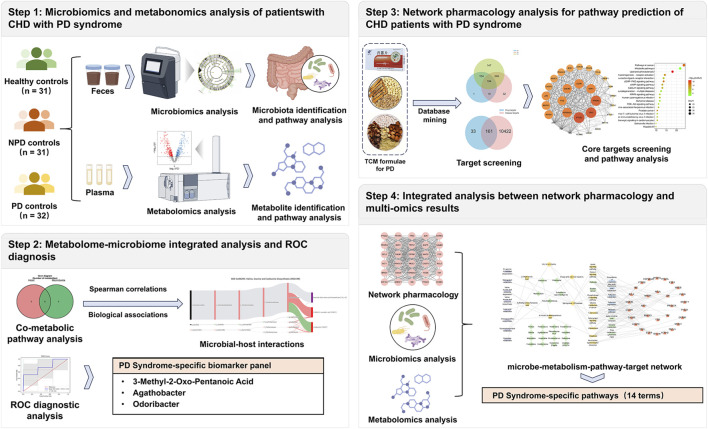
Flowchart of the multiomics and network pharmacology integration strategy of this study. First, clinical IR-CHD patients who were diagnosed with PD Syndrome by TCM physicians were enrolled. Microbiomics and metabolomics were employed to analyze fecal and serum samples from PD Syndrome patients, non-PD (NPD) Syndrome patients, and healthy controls (HC). Second, three typical TCM formulae clinically applied for PD Syndrome in CHD patients were selected for network pharmacology. Third, cross-validation between network pharmacology and multiomics results was conducted to characterize the biological basis of PD Syndrome in IR-CHD patients.

This study aimed to decipher the specific biological underpinnings of the PD Syndrome in patients with IR-CHD. To disentangle Syndrome-specific features from the general pathology of IR-CHD, we enrolled three distinct cohorts: IR-CHD patients with PD Syndrome (PD group), IR-CHD patients without PD syndrome (non-PD, NPD group), and healthy controls (HC). The inclusion of the NPD group served as a critical internal control to identify alterations attributable specifically to the PD Syndrome, rather than to IR-CHD *per se*. Subsequently, classic TCM formulae for the clinical treatment of IR-CHD patients with PD syndrome were selected to screen common drug targets for the construction of a protein‒protein interaction (PPI) network. Finally, the results from network pharmacology were cross-validated with the multiomics data (Figure 1). The results of this study could facilitate the modernization of TCM syndromes and provide a foundation for evaluating the efficacy of “syndrome differentiation and treatment” in the future.

## 2 Materials and methods

### 2.1 Participants

This study was conducted in compliance with the Declaration of Helsinki and the requirements of clinical trials by the Drug Administration Law of the People’s Republic of China in Guangdong Provincial Hospital of Traditional Chinese Medicine between April 2022 and November 2023.

The protocol and informed consent were reviewed and approved by the Ethics Committee of Guangdong Provincial Hospital of Chinese Medicine (No. BE2021-254-01). This trial was registered with the Chinese Clinical Trial Registry (ChiCTR) on 26 November 2020. (ID: ChiCTR2000040270). Informed consent was obtained from all participants, and their privacy rights were always preserved. All patients were enrolled by the Cardiology Clinic or Ward of Guangdong Provincial Hospital of Traditional Chinese Medicine or the surrounding communities in Guangzhou. Whether the study met the inclusion criteria (PD Syndrome of CHD) was determined by an expert (Xiaoqing Li, Deputy Chief Physician of Cardiovascular Medicine Dept at Guangdong Provincial Hospital of Traditional Chinese Medicine), with verification by two assistants (Xinyu Zhang and Haining Zhao).

### 2.2 Diagnostic criteria

Diagnostic criteria for CHD referred to the “Chinese Guidelines for Percutaneous Coronary Intervention (2016)” (Interventional Cardiology Group of Chinese Society of Cardiology of Chinese Medical Association et al., 2016): coronary angiography showed that any coronary artery stenosis was greater than 50%.

Diagnostic criteria for incomplete revascularization (IR) in CHD patients: The SYNTAX score published in the New England Journal of Medicine was used for calculation (Lee et al., 2009). In accordance with the ESC/EACTS Guidelines on Myocardial Revascularization in 2018 (Neumann et al., 2019), the SYNTAX residual score (rSS) was calculated by recording the baseline or preoperative SYNTAX score and postoperative SYNTAX score. rSS = 0 was defined as complete revascularization (CR), and rSS≥1 was defined as IR.

### 2.3 Diagnosis of TCM syndrome

According to the “Clinical diagnostic criteria for Phlegm-damp syndrome of coronary atherosclerotic heart disease” defined by the China Association of Chinese Medicine in 2017, the diagnostic criteria for Syndromes were determined as follows: the primary indicators (3 points/item) include pale swollen tongue with teeth marks, greasy tongue coating, and slippery tongue coating. The secondary indicators (2 points/item) included chest tightness and slippery or soggy pulses. Additional indicators (1 point/item) include heaviness of limbs, sticky mouth sensation, obesity, sticky stool, abdominal fullness, dull facial complexion, somnolence, and poor appetite.

When the cumulative score of the items in the “diagnostic criteria for Phlegm-damp syndrome” was ≥6, Phlegm-damp (PD) syndrome could be diagnosed. In the diagnostic process, three researchers (with at least one senior professional title) independently differentiated syndromes at the same time and then determined the types of syndromes together.

### 2.4 Inclusion criteria

Patients in the experimental group who meet the diagnostic criteria were potentially eligible for the study if they met the following criteria: (1) were aged 30–75 years and (2) were clearly diagnosed with coronary artery disease with incomplete coronary revascularization; (3) Patients who met or did not meet the diagnostic criteria for CHD with phlegm-damp syndrome in TCM after the doctor’s differentiation witness; (4) patients with stable vital signs, clear consciousness, and certain expression skills; and (4) voluntary submission of written informed consent prior to enrollment.

Healthy individuals in the control group who meet the diagnostic criteria were potentially eligible for the study if they met the following criteria: (1) were aged 30–75 years; and (2) voluntary submission of written informed consent prior to enrollment; (3) individuals did not meet the diagnostic criteria for PD Syndrome in TCM.

### 2.5 Exclusion criteria

Patients in the experimental group were excluded if they met one of the following criteria: (1) severe arrhythmia (either tachycardia or bradycardia) or severe valvular heart disease that may interfere with syndrome differentiation; (2) cardiogenic shock or significant heart failure (NYHA Class II-IV, or left ventricular ejection fraction ≤50%); (3) severe hepatic and/or renal dysfunction (serum alanine aminotransferase >3 times the upper limit of normal and/or glomerular filtration rate <45 mL/min/1.73 m^2^); (4) active bleeding, severe hematopoietic disorders, malignant tumors, or life expectancy less than 3 years; (5) use of gastric motility regulators, microecological drugs, antibiotics or traditional Chinese medicine preparations within the past 2 weeks; or (6) pregnant women, those planning to become pregnant, or lactating women.

Healthy individuals in the control group who met one of the following criteria: (1) Obesity (BMI 28 kg/m^2^); (2) abnormal liver and kidney functions (ALT and AST are twice the upper limit, or glomerular filtration rate <45 mL/min/1.73 cm^2^; (3) use of gastric motility regulators, microecological drugs, antibiotics or traditional Chinese medicine preparations within the past 2 weeks; (4) Previously diagnosed with major modern diseases, including those of the respiratory system, circulatory system, endocrine system, urinary system, nervous system, and digestive system.

### 2.6 Untargeted metabolomic profiling

The serum samples (100 μL), which were collected and pretreated, were examined via a Waters ACQUITY UPLC I-Class plus (Waters Corporation, Milford, USA). An ACQUITY UPLC HSS T3 column (1.8 μm, 2.1 × 100 mm) was used in both positive and negative modes. The original LC‒MS data were processed via Progenesis QI V2.3. OPLS-DA was employed to distinguish metabolites that differed between groups. A two-tailed Student’s t test was further conducted to verify whether the differences in metabolites between groups were statistically significant. Differentially abundant metabolites were selected on the basis of variable importance in projection (VIP) values greater than 1.0 and p values less than 0.05. (More detailed descriptions of the metabolomics process are provided in Supplementary Material).

### 2.7 Gut microbiome profiling

Collect fresh fecal samples from all participants and store them at −80 °C. Total genomic DNA from fecal samples was extracted via the MagPure Soil DNA LQ Kit. The extracted DNA was used as a template for PCR amplification of bacterial 16S rRNA genes with barcoded primers and Takara Ex Taq. For bacterial diversity analysis, the V3-V4 variable regions of the 16S rRNA genes were amplified with the universal primers 343F (5′-TACGGRAGGCAGCAG-3′) and 798R (5′-AGGGTATCTAATCCT-3′) (Nossa et al., 2010). The raw sequencing data were processed via the software to output the representative reads and the ASV abundance table. The representative read of each ASV was selected via the QIIME2 package. All representative reads were annotated and blasted against the Silva database via the q2-feature classifier with the default parameters. QIIME2 software was used for alpha and beta diversity analysis. The R package was subsequently used to analyze the significant differences between different groups via a t-test/Wilcoxon statistical test. The linear discriminant analysis effect size (LEfSe) method was used to compare the taxonomy abundance spectra. The functional composition of known microbial genes was predicted by utilising the PICRUSt2 software, with the objective of quantifying functional differences between various samples and groups (Douglas et al., 2020). (More detailed process descriptions of the gut microbiome are provided in Supplementary Material).

### 2.8 Network pharmacological analysis

For PD Syndrome in TCM of CHD, we selected 3 classic TCM formulae applied in the clinic for the treatment of this Syndrome. The treatment of each formula for the corresponding Syndrome was recommended in the Expert Consensus for Chinese Medicine Diagnosis and Treatment of Stable Angina Pectoris of CHD (Wang and Chen, 2018). The active compounds of each herb in the formulae and their corresponding targets were collected from the Encyclopedia of Traditional Chinese Medicine (ETCM) (http://www.tcmip.cn/ETCM/) (Xu et al., 2019) and the Traditional Chinese Medicine Systems Pharmacology Database and Analysis Platform (TCMSP) (https://www.tcmsp-e.com/tcmsp.php) (Ru et al., 2014). We filtered active compounds by OB ≥ 30% and DL ≥ 0.18 for ingredients from the TCMSP database, while ingredients from the ETCM database whose drug likeness grades were moderate or good were considered (QED≥0.49) to be active compounds. Disease targets were obtained by searching the GeneCards database (https://www.genecards.org/) (Stelzer et al., 2016) and OMIM (https://omim.org) via the keyword “coronary heart disease.”

Protein-protein interaction networks were constructed via the STRING database (https://cn.string-db.org/) (Franz et al., 2018). Metabolite‒gut microbiome association analysis was performed via the “deep MetOrigin analysis” module of MetOrigin 2.0 (https://metorigin.met-bioinformatics.cn/home/) (Yu et al., 2024). All networks were visualized and analyzed via Cytoscape software (Shannon et al., 2003).

### 2.9 Functional annotation analysis

Functional annotation analysis of genes was conducted via DAVID (https://davidbioinformatics.nih.gov/) (Huang et al., 2007) and the STRING platform. Bioinformatic analysis was performed via OECloud tools (https://cloud.oebiotech.com).

### 2.10 Statistical analysis

The data are expressed as the means ± standard deviations (means ± SD) or medians (P25-P75). One-way analysis of variance (ANOVA) was performed via IBM SPSS Statistics 27.0 software (IBM, Armonk, NY, US). To determine the significance, statistical tests were used to assess the distribution of variables and the nature of the data (Student’s t test, Mann-Whitney U test, Kruskal-Wallis test, Friedman test, F test). All of the comparison results were statistically significant (*p* < 0.05). GraphPad Prism 9.0 software (GraphPad Software, San Diego, California, US) was used for plotting.

## 3 Results

### 3.1 Characteristics of IR-CHD patients with phlegm-damp syndrome

A total of 94 participants, including IR-CHD patients with PD Syndrome (n = 32), NPD Syndrome (n = 31) and healthy controls (HC, n = 31), were enrolled at the Cardiology Clinic or Ward of Guangdong Provincial Hospital of Traditional Chinese Medicine or the surrounding communities in Guangzhou between April 2022 and November 2023. The demographic and clinical biochemical characteristics of the participants are listed in Table 1, while the personal history and comorbidities of the two disease groups are listed in Table 2. The disease group differed significantly from the HC group in terms of age, sex, and laboratory data, which may partly reflect the epidemiological and pathological features of the disease. However, there were no significant differences in age, sex, BMI, WHR, laboratory data, personal history, or comorbidities between the NPD and PD groups. Notably, the levels of TC and LDL-C in the blood samples of HC were greater than those in the blood samples of CHD patients. Elevated LDL and TC levels have been associated with an increased risk of CHD. Nevertheless, most healthy controls presented levels within the normal range. This discrepancy might be attributed to the relatively older age of the participants (most were approximately 60 years old) and the likelihood that individuals in the CHD group had already undergone lipid-lowering interventions.

**TABLE 1:** Demographic and clinical biochemical indicators of the participants.

Indicators	HC (n = 31)	NPD (n = 32)	PD (n = 32)	*P*
Basic information				
Age (year)	51.4 ± 1.8	62.5 ± 1.7	63.0 ± 1.8	*#
Female. n (%)	17 (54.8)	8 (25.8)	9 (28.1)	*#
BMI (kg/m2)	22.56 ± 0.70	23.02 ± 0.53	24.75 ± 0.95	n.s
WHR (%)	0.90 ± 0.01	0.92 ± 0.01	0.92 ± 0.01	n.s
Laboratory data				
ALT (U/L)	17 (13,25)	23 (14,39)	20 (16,32)	n.s
AST (U/L)	18.79 ± 1.22	22.00 ± 1.58	21.69 ± 1.73	n.s
Urea (mmol/L)	5.12 ± 0.25	6.15 ± 0.35	6.06 ± 0.24	*
Cr (μmol/L)	68.76 ± 3.34	82.87 ± 4.17	84.97 ± 3.58	*#
TG (mmol/L)	0.83 (0.55,1.23)	1.49 (1.05,2.11)	1.40 (1.06,2.13)	*#
TC (mmol/L)	4.50 (4.24,5.16)	3.76 (3.16,4.22)	4.02 (2.90,5.04)	*
HDL-C (mmol/L)	1.50 ± 0.057	1.30 ± 0.15	1.11 ± 0.041	#
LDL-C (mmol/L)	2.59 (2.21,3.18)	1.935 (1.12,2.23)	2.34 (1.47,3.39)	*
FBS(mmol/L)	4.84 (4.47,5.20)	5.82 (5.31,6.74)	5.98 (5.16,7.14)	*#
HCY(IU/L)	9.10 (6.66,10.15)	12.80 (10.18,15.50)	13.10 (9.75,15.95)	*#
BUA (μmol/L)	360.42 ± 20.82	377.48 ± 17.74	388.90 ± 18.54	n.s

BMI, body mass index; WHR, waist‒to-hip ratio; ALT, alanine transaminase; AST, aspartate transaminase; Cr, serum creatinine; TG, triglyceride; TC, total cholesterol; HDL-C, high-density lipoprotein cholesterol; LDL-C, low-density lipoprotein cholesterol; FBG, fasting blood glucose; HCY, homocysteine; BUA, blood uric acid.*. *p* < 0.05 for equality between HC, and NPD; #. *p* < 0.05 for equality between HC, and PD, patients; n.s. no significant differences among all groups. The Mann–Whitney U test was used to test for significant differences between the groups, *p* values were determined by one-way ANOVA, and the χ2 test, and the data are n (%), median (P25‒P75) or mean ± standard deviation (SD).

**TABLE 2:** Personal history and comorbidities of IR-CHD patients.

Indicators	PD (n = 32)	NPD (n = 32)	*P*
Duration (months)	12 (3,24)	24 (6,50)	0.066
rSS	8 (5,13)	7 (5,10)	0.476
Old myocardial infarction. n (%)	1 (3.13%)	3 (9.68%)	0.355
Arhythmia, n (%)	10 (31.25%)	6 (19.35%)	0.278
Hypertension, n (%)	7 (21.88%)	9 (29.03%)	0.514
Hyperlipidemia, n (%)	20 (62.5%)	17 (54.84%)	0.537
Diabetes. n (%)	12 (37.5%)	15 (48.39%)	0.383
Hyperhomocysteinemia, n (%)	3 (9.38%)	2 (6.45%)	1.000
Hyperuricemia, n (%)	5 (15.63%)	10 (32.26%)	0.121
Fatty liver. n (%)	5 (15.63%)	3 (9.68%)	0.708
Carotid arteriosclerosis. n (%)	4 (12.5%)	6 (19.35%)	0.509
Smoking history, n (%)	12 (37.5%)	9 (29.03%)	0.474
Alcohol consumption, n (%)	1 (3.01%)	3 (9.68%)	0.354
Family history of cardiovascular disease, n (%)	9 (28.13%)	10 (32.26%)	0.783

### 3.2 Microbiomics profiling of IR-CHD patients with PD syndrome

**FIGURE 2:**
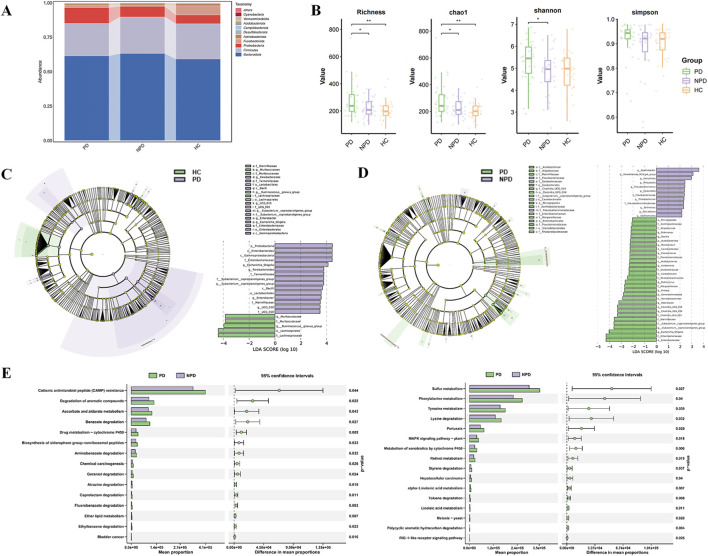
Analysis of the differential DGMs for PD Syndrome. **(A)** Relative abundance of species at the phylum level in each group. **(B)** Boxplot plot of the alpha diversity correlation. **(C,D)** LEfSe evolutionary branching diagram. The diameter of nodes was proportional to the relative abundance. The nodes in each layer represented phylum/class/order/family/genus from inside to outside, and the annotation of species markers in each layer represented phylum/class/order/family/genus from outside to inside. **(E)** Analysis of KEGG functional differences. The bar chart on the left represents pathways with significant differences in abundance between the two groups of samples. The box chart on the right represents the mean differences and confidence intervals between the two groups in the corresponding functional pathway. The dots represent the difference in mean proportions, and the line segments represent the confidence intervals.

On the basis of the species annotation results, we systematically analyzed the taxonomic abundance of the gut microbiota at multiple classification levels (class, order, family, and genus) across different experimental groups (Supplementary Figures S1A–D). The dominant phyla identified among the HC, NPD, and PD groups included *Bacteroidota*, *Proteobacteria*, *Firmicutes*, *Actinobacteriota*, and *Fusobacteriota* (Figure 2A). Compared with HC, the abundances of *Fusobacteria* and *Actinobacteria* decreased, whereas the abundances of *Proteobacteria* and *Bacteroidetes* increased in the disease groups. Additionally, the ratio of *Firmicutes* to *Bacteroidetes* (F/B) was reduced. Compared with the NPD group, the PD group presented greater abundances of Proteobacteria and Fusobacteria but lower abundances of *Bacteroidetes*, *Firmicutes*, and *Actinobacteria*. Furthermore, the F/B ratio was also lower in the PD group. Alpha diversity, assessed by the Richness, Chao1, Shannon, and Simpson indices, showed that both NPD and PD groups had significantly lower richness than HCs (Figure 2B). The PD group exhibited a higher Shannon index than the NPD group, indicating differences in community evenness. Beta diversity analysis revealed partial separation among groups. While PCoA showed overlapping clusters (Supplementary Figure S1E), NMDS indicated discernible segregation trends (Supplementary Figure S1F). Significant differences in overall microbiota composition were confirmed between HC and PD groups by Adonis and Anosim tests (Supplementary Tables S1, S2).

Using an LDA score threshold >2 and *p* < 0.05 as screening criteria to define the differential gut microbiota (DGMs), the evolutionary branch map from Linear discriminant analysis effect (LEfSe) analysis revealed that at the phylum level, *Firmicutes*, *Proteobacteria*, *Actinobacteria*, and *Fusobacteria* were significantly different between the HC and PD groups. Additionally, 15 genera were significantly different, with *Escherichia_Shigella* and *Ruminococcus* representing the two major evolutionary branches (Figure 2C). At the phylum level, *Firmicutes*, *Proteobacteria*, and *Actinobacteria* significantly varied between the PD and NPD groups, while 21 significantly diverse genera were identified (Figure 2D). The key biomes were distributed across three evolutionary branches: *Escherichia_Shigella*, *UCG_014*, and *Eubacterium_coprostanoligenes_group*. These results indicate that the gut microenvironment is substantially altered in IR-CHD patients and that PD syndrome may play a regulatory role in modulating the intestinal microenvironment of these patients.

These changes may alter the metabolism of the gut microbiota in IR-CHD patients with PD syndrome. Therefore, KEGG (Kyoto Encyclopedia of Genes and Genomes) functional prediction was further performed for the DGMs at the genus level between the NPD group and PD group. Significant alterations in 31 metabolic pathways were predicted (Figure 2E, *p* < 0.05), including amino acid metabolism (lysine degradation, aminobenzoate degradation, etc.), lipid metabolism (alpha‒linolenic acid metabolism, linoleic acid metabolism, etc.), exogenous substance metabolism and detoxification, signaling pathways, etc. Alpha‒linolenic acid (ALA) and linoleic acid (LA) are essential ω-3 and ω-6 polyunsaturated fatty acids, respectively. Research indicates that the balance of ω-6 and ω-3 fatty acids in the diet is a critical factor influencing cardiovascular health (Wijendran and Hayes, 2004). These findings suggest that PD syndrome may affect IR-CHD patients through metabolic functions mediated by the gut microbiota.

### 3.3 Metabolomics profiling of IR-CHD patients with PD syndrome

**FIGURE 3:**
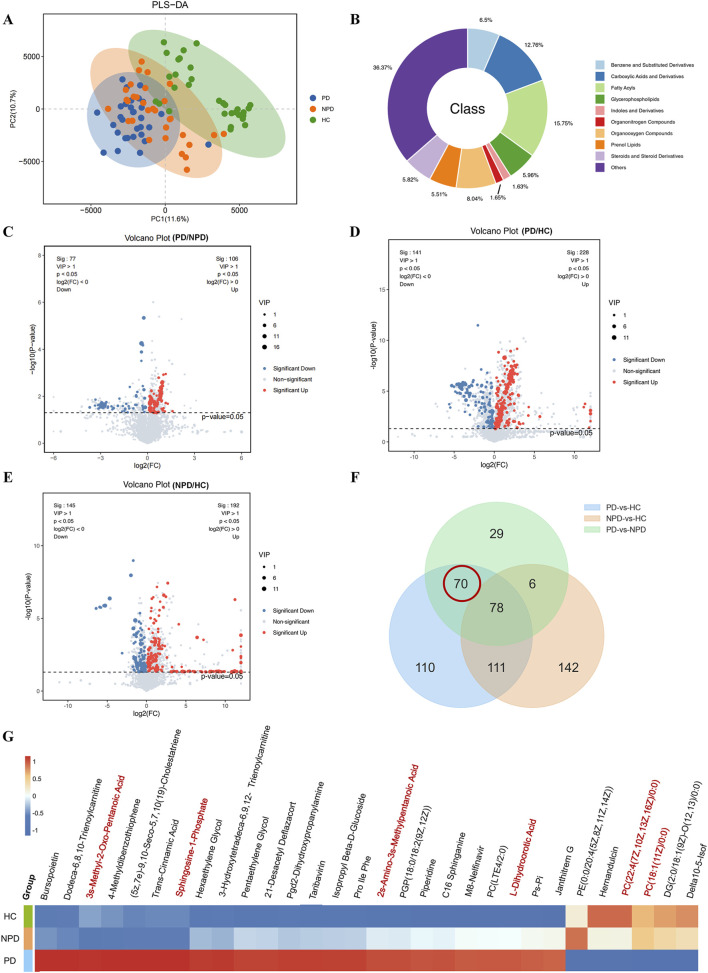
Metabolic analysis of IR-CHD patients with PD Syndrome. **(A)** PLS-DA plot. **(B)** Class classification chart for DMs. **(C–E)** Volcano plots of DMs between the PD and NPD, PD and HC, and NPD and HC groups. The criteria for screening were *p* value <0.05 and a VIP >1. **(F)** Venn diagram of the DMs filtered by the OPLS-DA model and univariate analysis. **(G)** DMs clustering heatmap. The abscissa represents the DMs, and the ordinate represents the group name. The color from blue to red indicates the mean expression abundance of DMs from low to high; that is, more red indicates a higher mean expression abundance of DMs. The red text DMs are specific to the serum of IR-CHD patients with PD Syndrome.

To investigate the metabolic features of PD Syndrome in IR-CHD patients, we performed non-targeted metabolomics using serum samples. On the PLS-DA score plot, the serum metabolomes of PD patients and NPD syndrome patients were clearly separated from those of HC (Figure 3A), but the separation between the PD group and NPD group was not obvious. On the OPLS-DA score plot, there was a clear separation of serum untargeted metabolomics between the PD, NPD, and HC groups (Supplementary Figures S2A–C). The identified differential metabolites (DMs) belonged to 10 categories, among which fatty acyls (15.75%), carboxylic acids and derivatives (12.76%) and organooxygen compounds (8.04%) were the three most influential metabolites (Figure 3B). Compared with those in the HC, 369 and 337 metabolites were significantly changed in the serum of PD patients and NPD patients, respectively. A total of 183 metabolites were significantly changed in the serum of PD patients and NPD patients, and all of them were upregulated (Figures 3C–E). DMs between groups were further analyzed via Venn diagrams. There were 148 overlapping DMs between the PD group and the HC group and between the PD group and the NPD group. After 78 DMs from the HC group and the NPN group were removed, 70 DMs specific to PD Syndrome patients were ultimately screened (Figure 3F; Supplementary Material; Supplementary Table S3). Hierarchical clustering showed that carboxylic acids and their derivatives, glycerophospholipids, and prenol lipids were the main metabolites (Figure 3G). These results suggest Syndrome-specific metabolic characteristics in IR-CHD patients.

**FIGURE 4:**
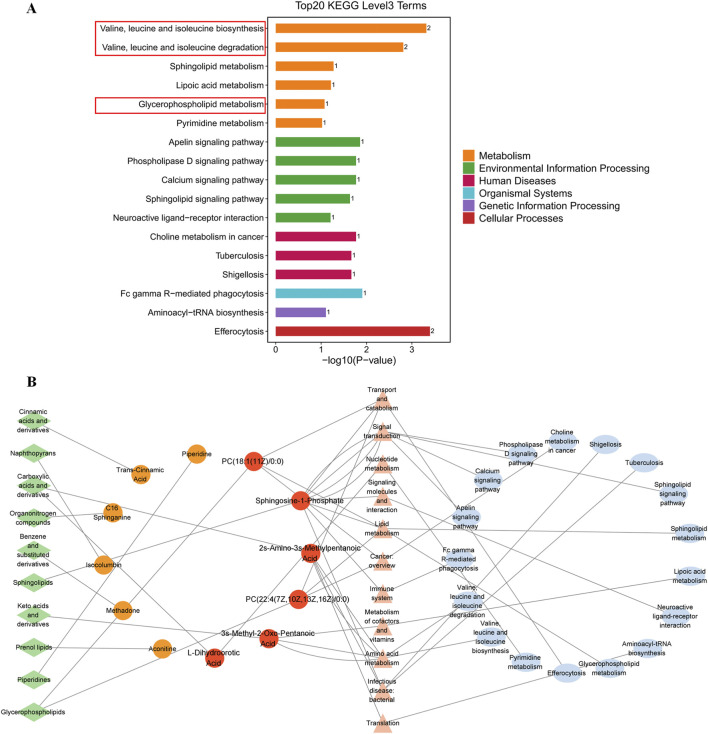
Functional enrichment and network analysis of DMs. **(A)** KEGG Level 3 distribution map of DMs. The abscis is the -log10 p value for each pathway, the ordinate is the different pathway name, the numbers on the columns are the number of DMs annotated to the pathway, and the different colors of the columns represent different KEGG Level 1 information. The metabolic pathways of microbial‒host interactions are shown in red boxes. **(B)** Differential metabolic network analysis of DMs enrichment in PD Syndrome. The green diamonds represent the class of metabolites, the blue ellipses represent the KEGG level 3 terms, the triangles represent the classification level 2 of the KEGG terms, the orange circles represent DMs with KEGG IDs, and the red circles represent DMs with KEGG IDs and *p* < 0.05.

On the basis of the 70 identified DMs, a total of 12 DMs with KEGG IDs, which were significantly altered in IR-CHD patients with PD Syndrome, were further analyzed. We then perform functional enrichment analysis for the DMs. Figure 4A shows that the DMs of the metabolites were enriched in 4 pathways of signal transduction (the sphingolipid signaling pathway, calcium signaling pathway, phospholipase D signaling pathway, and apelin signaling pathway), 2 pathways of lipid metabolism (glycerophospholipid metabolism and sphingolipid metabolism), and 2 pathways of amino acid metabolism (valine, leucine and isoleucine degradation, valine, and leucine and isoleucine biosynthesis). The analysis results considering the metabolome as a whole approach indicated that IR-CHD patients with PD syndrome were closely related to amino acid and lipid metabolism disorders. Next, Cytoscape software was used to map the DMs and differential metabolic pathway network (*p* < 0.05). 70 DMs and 17 KEGG metabolic pathways constituted the network diagram (Figure 4B). 6 DMs were identified on the basis of the metabolic pathways enriched and matched to differential metabolite IDs. These metabolites include 2 glycerophospholipids {PC[18:1 (11Z)/0:0] and PC [22:4 (7Z,10Z,13Z,16Z)/0:0]}, 2 sphingolipids [sphingomyelin 1-phosphate and SM (d18:0/16:1)], and 2 amino acids (2s-Amino-3s-Methylpentanoic Acid and L-Dihydroorotic Acid). These DMs are considered to be specific to the serum of IR-CHD patients with PD Syndrome.

### 3.4 Metabolome-microbiome integrated analysis and ROC analysis

**FIGURE 5:**
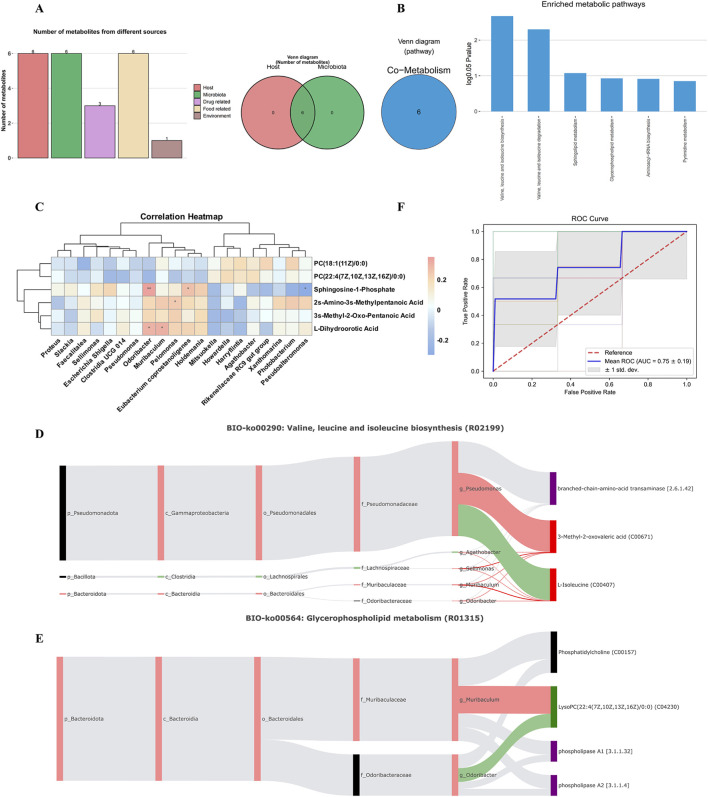
Metabolite-microbe interactions and ROC analysis. **(A)** Venn diagram and histogram tracking of DMs recalled by PD Syndrome. **(B)** Venn diagrams and histograms for enrichment analysis of metabolic pathways between the PD and NPD groups. **(C)** Correlation heatmap between DMs and DGMs at the genus level in the PD and NPD groups. *p < 0.05, **p < 0.01. **(D)** Sankey diagram of the valine, leucine and isoleucine biosynthesis reaction R02199. **(E)** Sankey diagram of the glycerophospholipid metabolism reaction R01315. **(F)** ROC diagnostic analysis of a biomarker panel (3-Methyl-2-Oxo-Pentanoic Acid, *Agathobacter*, *Odoribacter*) for PD Syndrome.

Despite advances in microbiome and metabolome tools, distinguishing host- from microbe-derived metabolites remains challenging. We therefore applied the “deep MetOrigin analysis” tool (MetOrigin 2.0), which integrates causal and homology analyses to elucidate microbial‒host interactions, to co-analyze the six syndrome-specific DMs and the DGMs at the genus level identified above. Traceability analysis (Figure 5A) revealed that the six Syndrome-specific DMs originated from host, microbial, drug, food, and environmental sources. Venn analysis indicated that all six DMs were common to both host and microbiota, suggesting that gut microbiota-driven metabolic disturbances are strongly implicated in the pathogenesis of PD Syndrome in IR-CHD patients.

The results of MPEA analysis via the MetOrigin platform revealed that 6 related metabolic pathways were paired with the co-metabolic pathway database of the host and microbiota between the PD and NPD groups (Figure 5B). Among them, metabolic pathways that have been identified as important metabolic pathways associated with PD syndromes in metabolomics studies, such as valine, leucine and isoleucine biosynthesis; valine, leucine and isoleucine degradation; sphingolipid metabolism; and glycerophospholipid metabolism, were also enriched. The results revealed significant disorders in amino acid and lipid metabolic pathways in PD syndrome patients, and their regulatory functions are related to both the host and the microbiota. Using the Sankey network analysis of the MetOrigin platform, Spearman correlations and biological associations between gender-level DGMs and DMs were calculated (Figure 5C) (Supplementary Material: Supplementary Table S4). 2s-Amino-3s-Methylpentanoic Acid and 3s-Methyl-2-Oxo-Pentanoic Acid were correlated with *Pelomonas* and *Pediococcus*, respectively. Sphingosine-1-Phosphate was correlated with 3 DGMs, and *Odoribacter* was negatively correlated with 2 DMs.

By analyzing the biological relationships between the microbiota and metabolites in important metabolic pathways, we conducted a visual analysis of the biological relationships. We identified 2 metabolic reactions (R02199 and R01315) that regulate the biosynthesis and degradation of valine, leucine, and isoleucine and glycerophospholipid metabolism, respectively, through which the microbiota may have a significant effect on PD syndrome. In R02199 metabolic reactions (Figure 5D), PD syndrome may significantly intervene in 5 bacterial communities at the genus level, activating branched-chain amino acid transaminases, leading to significant upregulation of 3-Methyl-2-oxovaleric acid and 2s-Amino-3s-Methylpentanoic Acid (L-Isoleucine). In R01315 metabolic reactions (Figure 5E), PD syndrome may activate phospholipase A1 and phospholipase A2 to metabolize PC(22:4 (7Z,10Z,13Z,16Z)/0:0), leading to a significant downregulation of PC(22:4 (7Z,10Z,13Z,16Z)/0:0) by significantly upregulating *Muribaculum* and *Odoribacter* at the genus level. In addition, *Agathobacter* at the genus level also activates lysophospholipase in the R02746 metabolic reaction, which is involved in PC[22:4 (7Z,10Z,13Z,16Z)/0:0] metabolism and shows a strong positive correlation with a decreasing trend. Notably, the abundance of *Agathobacter* was strongly positively correlated with decreasing levels of PC [22:4 (7Z,10Z,13Z,16Z)/0:0], suggesting that this bacterial genus may directly regulate the degradation or conversion of this phospholipid through enzymatic activity (Supplementary Figure S3A). PD syndrome may significantly intervene in 15 metabolic reactions through microbiota to regulate amino acid, sphingolipid, glycerophospholipid, pyrimidine and aminoacyl-tRNA metabolism. The above results indicate that PD Syndrome influences the metabolic level of IR-CHD patients, which significantly affects many metabolic functions. The reasons for these changes may be highly correlated with the host and microbial community.

Furthermore, receiver operating characteristic (ROC) diagnostic analysis was performed on 6 DMs and 9 DGMs with PD evidence specificity. 10-fold cross-validation and random forest modeling were applied to form a biomarker panel. In the metabolomics experiment, the area under the receiver operating curve (AUC) value of 1 DMs (3s-Methyl-2-Oxo-Pentanoic Acid) was 0.69 (Supplementary Figure S3B). In the microbiomics experiment, the AUC values of the 3 DGMs (*Agathobacter*, *Odoribacter*, and *Holdemania*) were 0.69, 0.64, and 0.62, respectively (Supplementary Figures S3C–E). This study demonstrated the potential of the aforementioned biomarkers to differentiate between PD patients and HC; however, the efficacy of this capability was found to be limited. Consequently, a combination of multiomics for composite characterization revealed that a biomarker panel could accurately distinguish PD patients from HC (Figure 5F). The biomarker panel comprised 1 DMs (3-Methyl-2-Oxo-Pentanoic Acid) and 2 DGMs (*Agathobacter, Odoribacter*), with an AUC value of 0.75. These findings indicate that the model has good performance, suggesting its applicability in adjunctive diagnosis and efficacy evaluation of PD symptoms.

### 3.5 Network pharmacology analysis of TCM formulae used to treat IR-CHD patients with PD syndrome in the clinic

**FIGURE 6:**
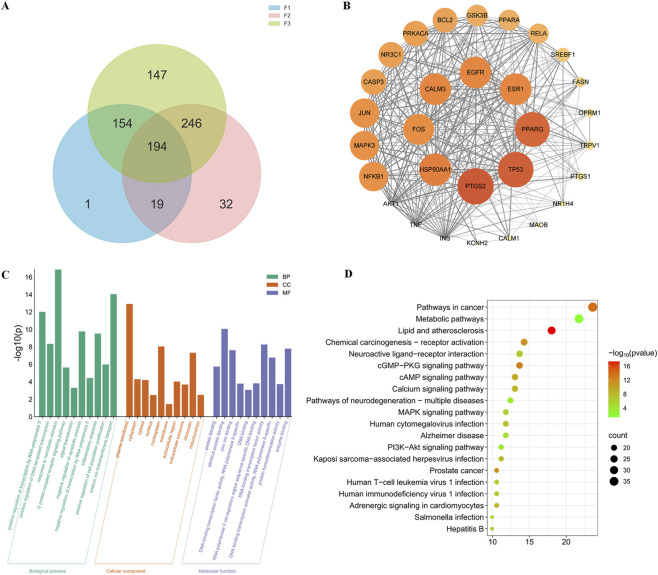
Network pharmacology analysis of the signature targets for the PD Syndrome formulae. **(A)** Overlapping targets of the 3 formulae for PD Syndrome. **(B)** Core targets of the PPI network formulae for CHD with PD Syndrome. **(C)** The top 10 of GO term enrichment analysis of the signature targets, based on the criteria of a minimum count of 2 and *p* < 0.05. **(D)** The top 20 of KEGG pathway enrichment analysis, based on the criteria of a minimum count of 2 and *p* < 0.05.

To investigate the features of the PD Syndromes from TCM formulae specifically used to treat it, we selected three TCM formulae recorded in the Expert Consensus for Chinese Medicine Diagnosis and Treatment of Stable Angina Pectoris of CHD and extensively used them for the treatment of the TCM Syndromes of CHD in the clinic. The formulae prescribed for PD Syndrome were Gualou Xiebai Banxia Decoction, Huanglian Wendan Decoction, and Danlou Tablet. For simplicity, we coded the formulae corresponding to PD Syndrome as F1, F2 and F3 (Table 3; see Supplementary Material; Supplementary Table S5 for the herbs in each formula). For each herb in the TCM formulae, we first collected its chemical ingredients and corresponding putative targets from the ETCM (Xu et al., 2019) and TCMSP (Ru et al., 2014) databases. We combined all the data to finally obtain 633 active compounds (Supplementary Table S6) with 788 putative targets (Table 3; Supplementary Table S7) for 18 different herbs in 3 TCM formulae. We considered the common targets of the three formulae for PD Syndrome as signature targets of the corresponding class of formulae. There were 194 signature targets for the classes of formulae (Figure 6A). The disease targets of CHD were retrieved from the GeneCards database and OMIM. Following the merging and removal of duplicate information, 10,583 disease targets of CHD were obtained. Finally, 161 common targets were identified via Venn analysis (Supplementary Figure S4A). The construction of the network was based on the STRING database. The protein‒protein interaction (PPI) network, which consisted of 158 nodes and 1,601 edges, was constructed via Cytoscape software. By setting the betweenness unDir, closeness unDir, and degree unDir parameter values, the network’s hub nodes are filtered out. (Figure 6B; Supplementary Figure S4B, Supplementary Table S8). The 30 core targets include: signal transduction (e.g., AKT1 and MAPK3), transcriptional regulation (e.g., NFKB1 and TP53), metabolic regulation (e.g., PPARA and FASN) and ion channels (e.g., KCNH2 and TRPV1). The majority of these targets are involved in central roles in cancer, metabolic diseases and inflammation.

**TABLE 3:** Number of herbs, potential bioactive compounds and corresponding putative targets for each TCM formula.

TCM formula	Formula code	# Herbs	#Activecompounds	#Putativetargets
Gualou xiebai banxia decoction	F1	3	68	368
Huanglian wendan decoction	F2	8	333	491
Danlou tablet	F3	10	311	821
Total		18	633	788

Next, the DAVID platform was employed to functionally annotate 161 intersecting targets. 385 biological process terms were significantly enriched by Gene Ontology (GO) enrichment analysis, included the G protein-coupled receptor signaling pathway, signal transduction, negative regulation of the apoptotic process, the inflammatory response, and positive regulation of cell population proliferation (Figure 6C). A total of 142 pathways terms were found to be significantly enriched by KEGG enrichment analysis, including the cGMP-PKG signaling pathway, the calcium signaling pathway, the cAMP signaling pathway, the adrenergic signaling in cardiomyocytes pathway, the PI3K-Akt signaling pathway, and the MAPK signaling pathway (Figure 6D). These pathways have been implicated in the mechanisms underlying coronary disease, with respect to factors such as plaque formation, vascular function, myocardial contraction, energy metabolism, and inflammation.

These results suggest that the targets and pathways regulated by Syndrome-specific prescriptions in PD may be the targets of Syndrome-specific dysfunction.

### 3.6 Integrated analysis between network pharmacology and multi-omics results

**FIGURE 7:**
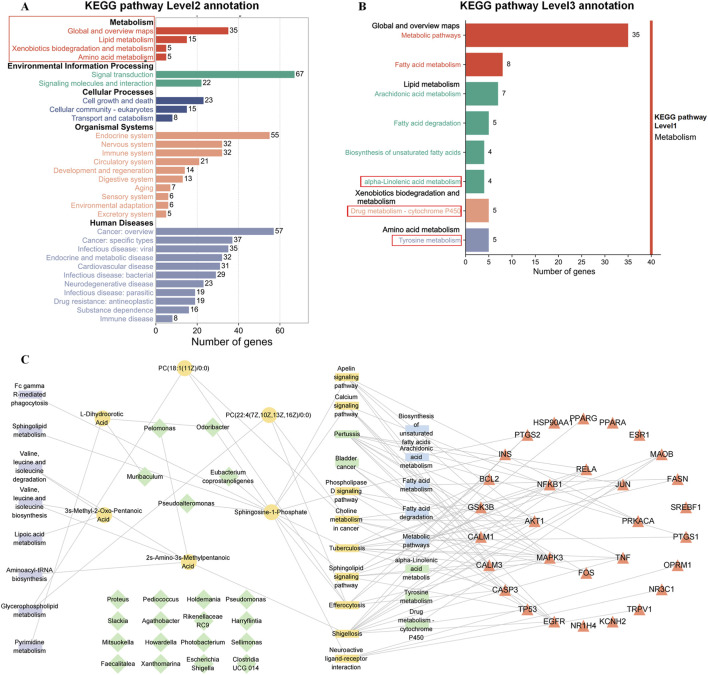
Integrated multiomics and network pharmacology analysis. **(A)** KEGG level 2 pathway enrichment results for network pharmacology. The red box shows the KEGG level 2 pathway terms of the KEGG level 1 pathway, namely, metabolism. **(B)** KEGG level 3 metabolism-related pathway enrichment results for network pharmacology. The red box illustrates the overlap between the KEGG level 3 pathway terms and the microbiome enrichment results. **(C)** The microbe-metabolism-pathway-target network. The green diamonds represent differential gut microbes; yellow circles denote differential metabolites; red triangles indicate core targets from network pharmacology; and rectangles represent KEGG pathways (green, shared by network pharmacology and microbiome; yellow, shared by network pharmacology and metabolome; blue, metabolism-related level 2 pathways unique to network pharmacology; and purple, pathways unique to metabolomics).

Our network pharmacological analysis indicated that targets of Syndrome-specific TCM formulae could be dysfunctional genes involved in the development of PD TCM Syndrome. Advances in biotechnology have enabled multiomics approaches, which integrate genomics, proteomics, metabolomics, and microbiomics to capture the complexity of biological systems and provide multidimensional data for mechanistic insight (Argelaguet et al., 2018). Therefore, an integrated analysis of network pharmacology, microbiomics, and metabolomics was conducted in this section. Considering that many KEGG pathways are involved in network pharmacology, we focused on metabolism-related pathways that play a role in the therapeutic mechanism of TCM. Therefore, the 142 enriched KEGG pathways were subjected to three-level classification to better identify the pathway affiliation overall (Figures 7A,B). The results revealed that there were 4 KEGG level 2 pathways and 8 KEGG level 3 pathways under KEGG pathway level 1 metabolism. Interestingly, we found that drug metabolism-cytochrome P450, tyrosine metabolism, and alpha-linolenic acid metabolism were also enriched in the microbiome. These results suggest that the mechanism of action of PD Syndrome formulae may be related to the regulation of metabolic pathways related to the gut microbiota.

To explore the mechanisms underlying the biological basis of PD Syndrome, we constructed a “microbe-metabolism-pathway-target” composite network on the basis of the core targets screened by network pharmacology, Syndrome-specific metabolites screened by metabolomics, and Syndrome-specific gut microbes screened by the microbiome and their enriched KEGG pathways (Figure 7C). The higher the node degree value is, the more connections there are to other nodes. In the network, Sphingosine-1-Phosphate (12°), 2s-Amino-3s-Methylpentanoic Acid (5°), and PC(22:4 (7Z,10Z,13Z,16Z)/0:0) (3°) were the 3 components with the highest degrees of association. MAPK3 (102°), AKT1 (84°), NFKB1 (72°), RELA (71°) and JUN (52°) were the 5 genes with the highest degree values. These genes may be the key metabolites and key genes for clinical TCM formulae of PD syndromes. In terms of mechanism interactions, we found that 6 DMs and network pharmacology had 9 shared KEGG pathway terms, whereas 31 DGMs and network pharmacology had 5 shared KEGG pathway terms (Supplementary Material: Supplementary Table S9). These KEGG pathways, which were identified through overlapping pathways, may be syndrome-specific pathways. Overall, these 14 pathways are involved mainly in metabolic regulation (choline, tyrosine, and lipid metabolism), cell signaling (calcium, phospholipase D, and apelin pathways) and neurological and drug metabolism-related processes, reflecting the interactions of multiple physiological and pathological processes. These findings indicated that the PD Syndrome-specific biomarkers screened by multiomics had a mutual connection in KEGG function enrichment with the pharmacological mechanism of the PD Syndrome formula, especially in amino acid and lipid metabolism. These findings suggest that gut microbiota-mediated metabolic disorders may be involved in IR-CHD patients with PD Syndrome, and that TCM formulae for the Syndrome can effectively intervene through a variety of mechanisms.

## 4 Discussion

There are still bottlenecks in the treatment of IR-CHD patients under the guidance of modern medicine. Many clinical evidence-based studies have confirmed that TCM has significant advantages in alleviating or improving the clinical symptoms and cardiac function of this type of patient, as well as in enhancing their quality of life (Ma et al., 2024; Chen et al., 2018). However, from the perspective of TCM theory, individualized and effective treatment plans are provided on the basis of the different syndromes of IR-CHD patients. PD TCM Syndrome, a key Syndrome in coronary heart disease patients, is closely related to their condition. Studies have reported that the etiology of CHD has evolved over time and across different populations. In the context of differences in physical constitution, the primary cause of CHD has shifted from chest Yang deficiency and cold congealing to phlegm turbidity, phlegm combined with stasis, and fire heat. This shift indicates a transition from cold to heat and from deficiency to excess (Lan et al., 2024). Therefore, distinguishing CHD from PD Syndromes differentiation CHD is one of the important therapeutic principles. This study integrated serum metabolomics, fecal microbiome analysis, and network pharmacology to characterize the differential serum metabolic and gut microbial profiles of PD Syndrome in IR-CHD patients. We identified potential Syndrome-specific biomarkers, explored gut microbiota-host metabolic interactions, and elucidated the biological underpinnings of PD Syndrome in this population.

There were distinct differences in laboratory test indicators of the 94 study participants. We found that the PD group had higher levels of red blood cells and hemoglobin than the non-PD certificate group did, reflecting the pathological basis of blood viscosity in CHD patients. This also aligns with the characteristics of Dampness as a pathogenic factor in traditional Chinese medicine theory, which is characterized by heaviness, turbidity, and viscosity. The results of the microbiome analysis at the phylum level revealed a tendency for a decrease in the ratio of *Firmicutes* to *Bacteroidetes* (F/B) in the two disease groups compared with that observed in HC. Furthermore, the F/B ratio was found to be lower in the PD group compared to the NPD group. The alteration in the F/B ratio is regarded as an indication of microbial dysbiosis (Stojanov et al., 2020; Bahar-Tokman et al., 2022; Remely and Haslberger, 2017), which has been linked to a decline in the production of short-chain fatty acids, notably butyrate, as well as protein metabolites such as histamine, and an accumulation of lipopolysaccharides. These phenomena have been demonstrated to elicit immune and inflammatory responses (Remely and Haslberger, 2017; An et al., 2023; De Luca and Shoenfeld, 2019; Mariat et al., 2009). However, the F/B ratio in patients with CHD remains controversial, possibly because various physiological and pathological conditions can influence the composition of the gut microbiota, resulting in many confounding factors. In this study, both PD patients and CHD patients presented decreased F/B ratios, providing supportive evidence for inflammatory immune pathological changes in both conditions. At the genus level, this study identified 21 DGMs involved in regulating amino acid and lipid metabolism through intergroup differences and functional enrichment analysis, suggesting that PD has a regulatory effect on the intestinal microenvironment of ICR-CHD patients. *Escherichia_Shigella*, *UCG_014*, and *Eubacterium_coprostanoligenes_group* were significantly different bacterial genera on the LEfSe evolutionary branch, suggesting that they have better functional synergy, environmental adaptability, and host-interaction capabilities (Chang et al., 2022). We speculate that they have better explanatory power in terms of functional contributions. *Escherichia_Shigella* has strong pathogenicity and is closely associated with numerous inflammatory diseases through the production of endotoxins. Reports have shown that *Escherichia_Shigella* can be used as a biomarker to distinguish coronary artery stenosis in patients with different degrees of CHD. Functional predictions suggest that it is associated with increased betaine biosynthesis and unsaturated fatty acid biosynthesis and decreased sphingolipid metabolism and primary bile acid biosynthesis (Yu et al., 2022). *Eubacterium* is one of the core genera of the human gut microbiota and plays a crucial role in nutrient metabolism and maintaining the intestinal balance. *Eubacterium* is one of the core genera of the human gut microbiota and plays a crucial role in nutrient metabolism and maintaining the intestinal balance. *Eubacterium_coprostanoligenes_group* (E. copr) is a subcategory of *Eubacterium*. Previous studies have reported that E. copr can metabolically convert dietary cholesterol into coprosterol in the jejunum and ileum, which is poorly absorbed in the gut, thereby reducing blood cholesterol levels (Bubeck et al., 2023; Ren et al., 1996; Hakansson and Molin, 2011). However, imbalanced blood cholesterol levels are a key risk factor for CHD. Therefore, the DGMs screened in this study may be potential biomarkers related to the efficacy of treatment for IR-CHD patients with PD syndrome, but the relevant molecular mechanisms still need further research.

LC-MS analysis of serum metabolites revealed that the 70 PD-specific DMs consisted primarily of lipids and lipid-like molecules (glycerophospholipids, fatty acyls, and prenol lipids) and amino acids. Further enrichment analysis revealed disruptions in metabolic pathways such as sphingolipid, glycerophospholipid, valine, leucine, and isoleucine pathways. Previous metabolomics studies (Gao-Song et al., 2019; Fan et al., 2016) have shown that patients with CHD exhibit severe metabolic disorders in pathways such as reduced phospholipid catabolism, increased amino acid metabolism, increased short-chain acylcarnitine, a reduced tricarboxylic acid cycle, and reduced synthesis of primary bile acids. Among these, glycerophospholipid metabolism disorders are the most prominent, and glycerophospholipid metabolism disorders are believed to constitute a key metabolic pathway in systemic immunity and low-grade inflammatory states (Zhu et al., 2022). Additionally, previous studies have shown that there are specific differential metabolites in CAD patients with phlegm and blood stasis and that lipid metabolism disorders occur more frequently in patients with phlegm (Cheng et al., 2015). Interestingly, the results of this study also revealed metabolic disorders in IR-CHD patients with PD Syndrome, specifically increased amino acid metabolism, glycerophospholipid metabolism disorders, and decreased fatty acid content, rather than alterations in the tricarboxylic acid or bile acid metabolism pathways. This finding likely suggests a unique metabolic profile for PD Syndrome.

Through joint analysis of the gut microbiota and metabolomics, we found that PD Syndrome in IR-CHD patients may be associated with significant upregulation of *Pseudomonas*, *Muribaculum* and *Odoribacter* and significantly downregulated *Agathobacter, Sellimonas* and *Faecalitalea* to promote 2s-Amino-3s-Methylpentanoic Acid (L-Isoleucine), 3s-Methyl-2-Oxo-Pentanoic Acid and PC[22:4 (7Z,10Z,13Z,16Z)/0:0] metabolism, thereby intervening in amino acid, glycerophospholipid, and sphingolipid metabolism. Branched-chain amino acids (BCAAs) consist of three essential amino acids: valine, leucine, and isoleucine. They are required for protein homeostasis, energy balance, and signaling pathways. Aberrant BCAAs catabolism has been observed in cardiovascular diseases (Zuo et al., 2023; Dziedzic et al., 2024). According to reports, impaired myocardial BCAA catabolism leads to BCAA accumulation and mTOR activation, contributing to cardiac dysfunction and remodeling (Wang et al., 2016). Additionally, propionyl-CoA generated during abnormal BCAA metabolism promotes platelet activation, which may drive microthrombosis and subsequent myocardial ischemia/infarction (Xu et al., 2020; Ou et al., 2021). The gut microbiota reshapes the host amino acid landscape and glucose homeostasis via efficiently metabolizing intestinal BCAAs. Oral supplementation with BCAAs can improve atherosclerosis (AS) by alleviating dyslipidemia and inflammation in ApoE^−/−^ mice. The regulation of the gut microbiota plays a crucial role in this process, particularly the significant alterations in *Faecalibaculum*, *Lachnospiraceae*, *Lactobacillus*, *Phascolarctobacterium*, and *Prevotellaceae_UCG-003*, which are similar to the findings of this study (Li et al., 2022). PC[22:4 (7Z,10Z,13Z,16Z)/0:0] is a lysophosphatidylcholine (LPC), a type of glycerophospholipid. As a serum biomarker for AS diagnosis, LPC exhibits broad biological effects. It is a key active component of oxidized low-density lipoprotein (ox-LDL) and contributes critically to AS progression by promoting macrophage phagocytosis and foam cell formation (Fernandez-Ruiz, 2022; Paapstel et al., 2018). Furthermore, LPC induces vascular endothelial cell (VEC) apoptosis through increased intracellular Ca^2+^, caspase-3 activation, and downregulation of survival factors such as cyclin D1, Bcl-xL, and Bcl-2 (Jung et al., 2017; Liu et al., 2020). LPC also triggers oxidative stress, enhances endothelial permeability, impairs endothelium-dependent vasodilation, and upregulates adhesion molecules and chemokines in VECs, leading to monocyte adhesion, macrophage chemotaxis, and vascular inflammation (Zhang et al., 2021; Rosenblat et al., 2005).

Finally, a “microbe-metabolism-pathway-target” network of PD patients was constructed by integrating network pharmacology, microbiome and metabolomics data. The key DMs (Sphingosine-1-Phosphate, 2s-Amino-3s-Methylpentanoic and PC[22:4 (7Z,10Z,13Z,16Z)/0:0)] and key genes (MAPK3, AKT1, NFKB1, RELA and JUN) involved in TCM prescription to exert personalized therapeutic effects on PD syndrome were identified. The important regulatory role of glycerophospholipid molecules was confirmed again. The method of integrated analysis of KEGG pathway terms of multiomics and network pharmacology is similar to the idea of “Syndrome Differentiation by Formula Prescription” in TCM theory. Research indicates that, on the one hand, the Clinical PD Syndrome formulae exert metabolic effects by modulating 9 signaling pathways, including the apelin signaling pathway, calcium signaling pathway, phospholipase D signaling pathway, and sphingolipid signaling pathway. On the other hand, the clinical PD syndrome formula may modulate the gut metabolic microenvironment through 5 metabolic pathways, such as tyrosine metabolism and alpha-linolenic acid metabolism. A network meta-analysis (Chao et al., 2023) revealed that Chinese herbal formulae for resolving phlegm and removing blood stasis, when combined with Western medicine, have a significant advantage in treating angina pectoris caused by phlegm and blood stasis interactions in CHD patients. The modified Zhishi Xiebai Guizhi Decoction (MZXGD) has great potential for improving the overall effective rate, shortening the duration of angina pectoris attacks, improving blood lipids, and reducing inflammatory factors. TCM can effectively intervene in CHD by regulating the composition of the gut microbiota and its metabolites, such as trimethylamine N-oxide (TMAO), short-chain fatty acids (SCFAs), and bile acids (BAs). Therefore, our study provides new insights into the regulation of the intestinal microenvironment in IR-CHD patients with PD syndrome by TCM.

It is important to acknowledge the limitations inherent in this research. First, owing to limitations in research funding and time, the size of the clinical sample was relatively small. There is room for improvement in sample size to cover a broader research population. Second, as an observational study, there are several potential confounding factors to be considered. It is important to note that there is an incomplete matching of demographic characteristics (e.g., age, sex) between the two disease groups and the HC group. This is reflective of the disease’s epidemiology, but it may influence the interpretation of comparisons involving the HC group. Although the inclusion of the NPD group as an internal control did facilitate the isolation of Syndrome-specific feature, it remains challenging to entirely control for all potential confounders (e.g., diet, medication, lifestyle) that affect metabolomics and microbiome analysis. The validation of these findings in future studies with more strictly matched cohorts is warranted. Third, the elements and objectives of the formulae are obtained from databases. Despite the integration of data from the majority of currently available TCM databases and the study of multiple TCM formulae to increase the reliability of targets, the inherent quality of the databases may introduce variability in the results.

## 5 Conclusion

This study demonstrated that PD Syndrome formulae may act on key proteins such as MAPK3, AKT1, NFKB1, RELA, and JUN, regulating signaling pathways (apelin, calcium, phospholipase D, and sphingolipid) and metabolism (choline, tyrosine, and alpha‒linolenic acid), thereby influencing amino acid, glycerophospholipid, and sphingolipid metabolism, and aminoacyl‒tRNA biosynthesis involving the gut microbiota, thereby improving PD syndrome in IR-CHD patients. However, the specific molecular mechanisms underlying gut–host–microbe interactions and this syndrome require further investigation. This study contributes to our understanding of the biological basis of TCM syndromes from a modern systems biology perspective, advancing the modernization of TCM theory and application.

## Data Availability

The 16S rRNA amplicon sequencing data generated in this study were submitted to the NCBI Sequence Read Archive (SRA) under BioProject accession number PRJNA1285355. The raw metabolomics data reported in this paper have been deposited in OMIX, China National Center for Bioinformation/Beijing Institute of Genomics, Chinese Academy of Sciences, accession number OMIX011165.
