# Supplementary material for: The gut microbiota remodes amino acid and lipid metabolism in incomplete revascularization of CHD with phlegm-dampness syndrome: an integrated multiomics and network pharmacology study
Source: Front Mol Biosci. 2026 Mar 9;13:1748007. doi: 10.3389/fmolb.2026.1748007 (PMC13006993; doi:10.3389/fmolb.2026.1748007)
Supplement: Supplementary file 1 [file Supplementaryfile1.docx]

Supplementary Material

# Supplementary Data

## Untargeted Metabolomics profiling:

### LC-MS/MS analysis

The metabolomic data analysis was performed by Shanghai Luming biological technology co., LTD (Shanghai, China).

An ACQUITY UPLC I-Class plus（Waters Corporation，Milford, USA） fitted with Q-Exactive mass spectrometer equipped with heated electrospray ionization (ESI) source (Thermo Fisher Scientific, Waltham, MA, USA) was used to analyze the metabolic profiling in both ESI positive and ESI negative ion modes. An ACQUITY UPLC HSS T3 column (1.8 μm, 2.1 × 100 mm) were employed in both positive and negative modes. The binary gradient elution system consisted of (A) water (containing 0.1 % formic acid, v/v) and (B) acetonitrile (containing 0.1 % formic acid, v/v) and separation was achieved using the following gradient: 0.01 min, 5% B; 2min, 5% B; 4min, 30% B; 8min, 50% B; 10min, 80% B; 14min, 100% B; 15 min, 100% B; 15.1 min, 5% and 16 min, 5%B. The flow rate was 0.35 mL/min and column temperature was 45℃. All the samples were kept at 10℃ during the analysis. The injection volume was 3 μL.

The mass range was from m/z 100 to 1,000. The resolution was set at 70,000 for the full MS scans and 17500 for HCD MS/MS scans. The Collision energy was set at 10, 20 and 40 eV. The mass spectrometer operated as follows: spray voltage, 3800 V (+) and 3200 V (−); sheath gas flow rate, 35 arbitrary units; auxiliary gas flow rate, 8 arbitrary units; capillary temperature, 320°C; Aux gas heater temperature, 350°C; S-lens RF level, 50.

### Data Preprocessing and Statistical Analysis

The original LC-MS data were processed by software Progenesis QI V2.3 (Nonlinear, Dynamics, Newcastle, UK) for baseline filtering, peak identification, integral, retention time correction, peak alignment, and normalization. Main parameters of 5 ppm precursor tolerance, 10 ppm product tolerance, and 5% product ion threshold were applied. Compound identification were based on precise mass-to-charge ratio (M/z), secondary fragments, and isotopic distribution using The Human Metabolome Database (HMDB), Lipidmaps (V2.3), Metlin, and self-built databases. The extracted data were then further processed by removing any peaks with a missing value (ion intensity = 0) in more than 50% in groups, by replacing zero value by half of the minimum value, and by screening according to the qualitative results of the compound. Compounds with resulting scores below 36 (out of 60) points were also deemed to be inaccurate and removed. A data matrix was combined from the positive and negative ion data.

The matrix was imported in R to carry out Principle Component Analysis (PCA) to observe the overall distribution among the samples and the stability of the whole analysis process. Orthogonal Partial Least-Squares-Discriminant Analysis (OPLS-DA) and Partial Least-Squares-Discriminant Analysis (PLS-DA) were utilized to distinguish the metabolites that differ between groups. To prevent overfitting, 7-fold cross-validation and 200 Response Permutation Testing (RPT) were used to evaluate the quality of the model. V ariable Importance of Projection (VIP) values obtained from the OPLS-DA model were used to rank the overall contribution of each variable to group discrimination. A two-tailed Student’s T-test was further used to verify whether the metabolites of difference between groups were significant. Differential metabolites were selected with VIP values greater than 1.0 and p-values less than 0.05. Differential metabolites were further used to for KEGG pathway (http://www.genome.jp/kegg/) enrichment analysis.

# 16S rRNA amplicon sequencing experimental method

## DNA extraction and amplification

Total genomic DNA was extracted using MagPure Soil DNA LQ Kit (Magan) following the manufacturer’s instructions. DNA concentration and integrity were measured with NanoDrop 2000 (Thermo Fisher Scientific, USA) and agarose gel electrophoresis. Extracted DNA was stored at -20°C until further processing. The extracted DNA was used as template for PCR amplification of bacterial 16S rRNA genes with the barcoded primers and Takara Ex Taq (Takara). For bacterial diversity analysis, V3-V4 variable regions of 16S rRNA genes was amplified with universal primers 343F (5’-TACGGRAGGCAGCAG-3’) and 798R (5’-AGGGTATCTAATCCT-3’).

## Library construction and sequencing

## The Amplicon quality was visualized using agarose gel electrophoresis. The PCR products purified with AMPure XP beads (Agencourt) and amplified for another round of PCR. After purified with the AMPure XP beads again, the final amplicon was quantified using Qubit dsDNA Assay Kit (Thermo Fisher Scientific,USA). The concentrations were then adjusted for sequencing. Sequencing was performed on an Illumina NovaSeq 6000 with 250 bp paired-end reads. (Illumina Inc., San Diego, CA; OE Biotech Company; Shanghai, China).

# 16S rRNA amplicon sequencing analysis process

## Bioinformatic analysis

The library sequencing and data processing were conducted by OE biotech Co., Ltd. (Shanghai, China). Raw sequencing data were in FASTQ format. Paired-end reads were then preprocessed using Cutadapt software to detect and cut off the adapter. After trimming, paired-end reads were filtering low quality sequences, denoised, merged and detect and cut off the chimera reads using DADA2 with the default parameters of QIIME2 (2020.11). At last, the software output the representative reads and the ASV abundance table. The representative read of each ASV was selected using QIIME2 package. All representative reads were annotated and blasted against Silva database (Version 138) using q2-feature-classifier with the default parameters.

QIIME2 software was used for alpha and beta diversity analysis. The microbial diversity in samples was estimated using the alpha diversity that include Chao1 index and Shannon index. The unweighted Unifrac distance matrix performed by R package was used for unweighted Unifrac Principal coordinates analysis (PCoA) to estimate the beta diversity. Then the R package was used to analyze the significant differences between different groups using T test/Wilcoxon statistical test.The linear discriminant analysis effect size (LEfSe) method was used to compare the taxonomy abundance spectrum.

**Table S1 Adonis analysis of gut microbiota**

| **Group** | **R^2^** | ***P*** |
| --- | --- | --- |
| HC / PD | 0.02238 (0.97762) | 0.041* |
| HC / NPD | 0.01938 (0.98062） | 0.156 |
| PD / NPD | 0.01437 (0.98563) | 0.716 |

R^2^ is the ratio of the grouped variance to the total variance, indicating the degree of explanation of the differences between samples by the sample groupings, with larger values representing a higher degree of explanation of the differences by the groupings. The values in parentheses indicate the corresponding residual values. **p* < 0.05, ***p* < 0.01, ****p* < 0.001.

**Table S2 Anosim analysis of gut microbiota**

| **Group** | **R^2^** | ***P*** |
| --- | --- | --- |
| HC / PD | 0.046204797708557066 | 0.035* |
| HC / NPD | 0.021875746714456416 | 0.136 |
| PD / NPD | 0.000167835923601149 | 0.442 |

R^2^ > 0 indicates that the between-group difference is greater than the within-group difference, and R^2^ < 0 the between-group difference is less than the within-group difference. **p* < 0.05, ***p* < 0.01, ****p* < 0.001.

**Table S3 70 DMs specific of PD syndrome**

| **Ion mode** | **Metabolites** | **KEGG** | **Class** | **Score** |
| --- | --- | --- | --- | --- |
| neg | PC(18:1(11Z)/0:0) | C04230 | Glycerophospholipids | 79.3 |
| pos | Sphingosine-1-Phosphate | C06124 | Sphingolipids | 69.8 |
| neg | PC(22:4(7Z,10Z,13Z,16Z)/0:0) | C04230 | Glycerophospholipids | 63.7 |
| pos | (5z,7e)-9,10-Seco-5,7,10(19)-Cholestatriene | | Steroids and steroid derivatives | 63.3 |
| neg | PE(0:0/20:4(5Z,8Z,11Z,14Z)) | | Glycerophospholipids | 63 |
| pos | Hernandulcin | | Prenol lipids | 62.8 |
| pos | 2s-Amino-3s-Methylpentanoic Acid | C00407 | Carboxylic acids and derivatives | 58.9 |
| pos | Piperidine | C01746 | Piperidines | 58.8 |
| pos | Hexaethylene Glycol | | Organooxygen compounds | 52.4 |
| pos | C16 Sphinganine | C13915 | Organonitrogen compounds | 50.1 |
| pos | 3-O-(2-O-(2e-Decenoyl)-Alpha-L-Rhamnopyranosyl)-3-Hydroxydecanoic Acid | | Fatty Acyls | 48.4 |
| pos | Ibuprofen Piconol | | Prenol lipids | 48.2 |
| pos | 1-(2-Hydroxyethyl)-2-Hydroxymethyl-5-Nitroimidazole | | Azoles | 47.4 |
| pos | Methadone | C07163 | Benzene and substituted derivatives | 46.3 |
| pos | DG(2:0/18:1(9Z)-O(12,13)/0:0) | | Unclassified | 45.8 |
| pos | Pentaethylene Glycol | | Organooxygen compounds | 45.8 |
| pos | Isopropyl Beta-D-Glucoside | | Organooxygen compounds | 45.6 |
| pos | Okooa-Pc |  | Glycerophospholipids | 45.6 |
| pos | Cer(d18:1/6 keto-PGF1alpha) | | Unclassified | 45.6 |
| pos | Delta10-5-Isof | | Organooxygen compounds | 44.9 |
| pos | Mrz 2266 Bs | | 6,7-benzomorphans | 44.8 |
| pos | Pro Ile Phe | | Unclassified | 43.9 |
| neg | 3s-Methyl-2-Oxo-Pentanoic Acid | C00671 | Keto acids and derivatives | 43.1 |
| pos | Trans-Cinnamic Acid | C10438 | Cinnamic acids and derivatives | 42.7 |
| pos | Aplaviroc |  | Piperidines | 41.9 |
| pos | Bursopoietin | | Carboxylic acids and derivatives | 41.7 |
| pos | 3-Hydroxytetradeca-6,9,12-Trienoylcarnitine | | Fatty Acyls | 41.5 |
| pos | PGP(18:0/18:2(9Z,12Z)) | | Glycerophospholipids | 41.5 |
| pos | Isocolumbin | C17508 | Naphthopyrans | 41.4 |
| pos | Galactoarabinan | | Organooxygen compounds | 41.4 |
| pos | 21-Desacetyl Deflazacort | | Steroids and steroid derivatives | 41.3 |
| pos | Taribavirin |  | Triazole ribonucleosides and ribonucleotides | 41.2 |
| pos | 1,1,2-Trimethyl-3,5-Bis(1-Methylethenyl)Cyclohexane | | Prenol lipids | 41 |
| pos | L-Dihydroorotic Acid | C00337 | Carboxylic acids and derivatives | 40.9 |
| neg | 4-Methyldibenzothiophene | | Benzothiophenes | 40.5 |
| pos | Biricodar |  | Carboxylic acids and derivatives | 40.3 |
| pos | Posaconazole | | Diazinanes | 40.3 |
| pos | Apramycin | | Organooxygen compounds | 39.8 |
| pos | Dodeca-6,8,10-Trienoylcarnitine | | Fatty Acyls | 39.5 |
| pos | PC(LTE4/2:0) | | Unclassified | 39.5 |
| pos | Ps-Pi |  | Glycerophospholipids | 39.3 |
| pos | L-Prolinamide, 5-Oxo-L-Prolyl-L-Norvalyl- | | Carboxylic acids and derivatives | 39 |
| pos | Aconitinum | | Prenol lipids | 38.8 |
| pos | Simulanoquinoline | | Benzophenanthridine alkaloids | 38.8 |
| pos | Corchorusoside B | | Steroids and steroid derivatives | 38.8 |
| pos | Janthitrem G | | Naphthopyrans | 38.6 |
| pos | Aconitine | C06091 | Prenol lipids | 38.5 |
| pos | PGP(a-13:0/i-12:0) | | Glycerophospholipids | 38.4 |
| pos | 4-[2-Acetylamino-2-(1-Biphenyl-4-Ylmethyl-2-Oxo-Azepan-3-Ylcarbamoyl)-Ethyl]-2-Formyl-Benzoic Acid | | Carboxylic acids and derivatives | 38.4 |
| pos | N-Lauroyl Tryptophan | | Carboxylic acids and derivatives | 38.4 |
| pos | Cepharanthine | | Unclassified | 38.2 |
| pos | M8-Nelfinavir | | Carboxylic acids and derivatives | 38.1 |
| pos | Aspacoside E | | Steroids and steroid derivatives | 38.1 |
| pos | 24-Acetyl- 25-Cinnamoylvulgaroside | | Prenol lipids | 38 |
| pos | Ppgpp |  | Purine nucleotides | 37.9 |
| pos | N-Formyl-Nle-Leu-Phe | | Carboxylic acids and derivatives | 37.6 |
| pos | PGP(i-12:0/PGE2) | | Unclassified | 37.6 |
| pos | PI(18:1(11Z)/LTE4) | | Unclassified | 37.5 |
| pos | PA(14:1(9Z)/6 keto-PGF1alpha) | | Unclassified | 37.3 |
| pos | Lisuride |  | Quinolines and derivatives | 37.3 |
| pos | 5-Valyl Angiotensin Ii | | Carboxylic acids and derivatives | 37 |
| pos | PA(8:0/20:3(5Z,8Z,11Z)-O(14R,15S)) | | Unclassified | 36.9 |
| pos | Satavaptan | | Benzene and substituted derivatives | 36.8 |
| pos | PGP(i-12:0/20:4(6Z,8E,10E,14Z)-2OH(5S,12R)) | | Unclassified | 36.7 |
| pos | PGP(18:2(9Z,11Z)/6 keto-PGF1alpha) | | Unclassified | 36.7 |
| pos | 3-O-Protocatechuoylceanothic Acid | | Prenol lipids | 36.6 |
| pos | Pgd2-Dihydroxypropanylamine | | Fatty Acyls | 36.2 |
| pos | Cholesteryl Glucoside | | Steroids and steroid derivatives | 36.1 |
| pos | Calcium Caprate | | Fatty Acyls | 36 |
| pos | MGDG(18:4(6Z,9Z,12Z,15Z)/18:4(6Z,9Z,12Z,15Z)) | | Fatty Acyls | 36 |

Score scoring description: a full score of 80 points, primary mass spectrometry accurate molecular weight matching (20 points), secondary mass spectrometry fragment matching (20 points), isotope distribution matching (20 points), retention time matching (20 points), in general, the higher the score, the more accurate qualitative.

**Table S4 Spearman analysis of correlation between DMs and gender-level DGMs**

| **DMs** | **DGMs** | **Correlation** | ***p*-value** | **AdjPvalue** | **Significance** |
| --- | --- | --- | --- | --- | --- |
| 2s-Amino-3s-Methylpentanoic Acid | Pelomonas | 0.2482 | 0.0499 | 0.1611 | * |
| 3s-Methyl-2-Oxo-Pentanoic Acid | Pediococcus | 0.3138 | 0.0123 | 0.0736 | * |
| Sphingosine-1-Phosphate | Eubacterium coprostanoligenes | 0.2684 | 0.0334 | 0.2007 | * |
| Sphingosine-1-Phosphate | Odoribacter | 0.3523 | 0.0046 | 0.0278 | ** |
| Sphingosine-1-Phosphate | Pseudoalteromonas | -0.2949 | 0.0190 | 0.1138 | * |
| L-Dihydroorotic Acid | Odoribacter | 0.3067 | 0.0145 | 0.0434 | * |
| L-Dihydroorotic Acid | Muribaculum | 0.3080 | 0.0141 | 0.0844 | * |

**p* < 0.05, ***p* < 0.01, ****p* < 0.001.

**Table S5 Herbs in each TCM formula**

| **TCM formula** | **Formula code** | **# Herbs (English, Latin, Chinese Pinyin)** |
| --- | --- | --- |
| Gualou Xiebai Banxia Decoction | F1 | Trichosanthis Fructus, *Trichosanthes kirilowii*, Gua Lou |
|  |  | Allii Macrostemonis Bulbus, *Allium Azureum Ledeb*, Xie Bai |
|  |  | Pinelliae Rhizoma, *Arum Ternatum Thunb*, Ban Xia |
| Huanglian Wendan Decoction | F2 | Coptidis Rhizoma, *Coptis chinensis*, Huang Lian |
|  |  | Bambusae Caulis in Taeniam, *Phyllostachys spp*, Zhu Ru |
|  |  | Aurantii Fructus Immaturus, *Citrus aurantium*, Zhi Shi |
|  |  | Pinelliae Rhizoma, *Arum Ternatum Thunb*, Ban Xia |
|  |  | Citri Reticulatae Pericarpium, *Citrus reticulata*, Chen Pi |
|  |  | Glycyrrhizae Radix et Rhizoma, *licorice*, Gan Cao |
|  |  | Zingiberis Rhizoma Recens, *Zingiber Officinale Roscoe*, Sheng Jiang |
|  |  | Poria, *Poria Cocos(Schw.) Wolf*, Fu Ling |
| Danlou Tablet | F3 | Trichosanthis Fructus, Trichosanthes kirilowii, Gua Lou |
|  |  | Allii Macrostemonis Bulbus, *Allium Azureum Ledeb*, Xie Bai |
|  |  | Puerariae Lobatae Radix, *Radix Puerariae*, Ge Gen |
|  |  | Chuanxiong Rhizoma, *Ligusticum chuanxiong*, Chuan Xiong |
|  |  | Salviae Miltiorrhizae Radix et Rhizoma, *Radix Salviae*, Dan Shen |
|  |  | Paeoniae Radix Rubra, *Radix Paeoniae Rubra*, Chi Shao |
|  |  | Alismatis Rhizoma, *Alisma Orientale (Sam.) Juz*, Ze Xie |
|  |  | Astragali Radix, *Hedysarum Multijugum Maxim*, Huang Qi |
|  |  | Drynariae Rhizoma, *Drynaria fortunei*, Gu Sui Bu |
|  |  | Curcumae Radix, *Curcuma aromatica*, Yu Jin |

**Table S6. Active compounds in each formula.**

See the Excel file with the same name for details.

**Table S7. Putative targets in each formula.**

See the Excel file with the same name for details.

**Table S8. PPI network of 30 key proteins**

| **Betweenness unDir** | **Closeness unDir** | **Degree unDir** | **name** | **selected** | **shared name** |
| --- | --- | --- | --- | --- | --- |
| 185.0654336 | 0.002237136 | 4 | ABAT | TRUE | ABAT |
| 304.6414243 | 0.002873563 | 18 | ACOX1 | FALSE | ACOX1 |
| 138.867719 | 0.002188184 | 5 | ALDH5A1 | TRUE | ALDH5A1 |
| 1150.471691 | 0.003164557 | 23 | MAOB | FALSE | MAOB |
| 0 | 0.001675042 | 2 | OGDH | TRUE | OGDH |
| 0 | 0.001848429 | 1 | ABCC5 | TRUE | ABCC5 |
| 315.9928979 | 0.002597403 | 5 | LTB4R2 | FALSE | LTB4R2 |
| 19.29653745 | 0.002898551 | 8 | ABCC8 | TRUE | ABCC8 |
| 383.3611432 | 0.00297619 | 21 | KCNH2 | FALSE | KCNH2 |
| 710.4868101 | 0.003831418 | 62 | PPARG | FALSE | PPARG |
| 1969.416201 | 0.003717472 | 58 | CALM3 | FALSE | CALM3 |
| 1180.702216 | 0.003610108 | 50 | PRKACA | FALSE | PRKACA |
| 88.05015103 | 0.003184713 | 29 | HNF4A | FALSE | HNF4A |
| 939.7571035 | 0.003717472 | 59 | HSP90AA1 | FALSE | HSP90AA1 |
| 2086.13556 | 0.004098361 | 75 | INS | FALSE | INS |
| 2.139005907 | 0.0025 | 6 | SLC8A1 | TRUE | SLC8A1 |
| 1.436279203 | 0.002985075 | 21 | ABL1 | FALSE | ABL1 |
| 190.4563792 | 0.003424658 | 42 | MAPK1 | FALSE | MAPK1 |
| 77.65585231 | 0.003401361 | 39 | NFKBIA | FALSE | NFKBIA |
| 277.1923654 | 0.003676471 | 55 | NFKB1 | FALSE | NFKB1 |
| 170.6629179 | 0.003355705 | 36 | IL2 | FALSE | IL2 |
| 59.0051693 | 0.003236246 | 31 | CDK2 | FALSE | CDK2 |
| 664.5106763 | 0.003745318 | 63 | TP53 | FALSE | TP53 |
| 716.9702812 | 0.003703704 | 58 | EGFR | FALSE | EGFR |
| 685.789529 | 0.003690037 | 57 | FOS | FALSE | FOS |
| 229.1180348 | 0.003676471 | 50 | CASP3 | FALSE | CASP3 |
| 295.1670789 | 0.003584229 | 45 | GSK3B | FALSE | GSK3B |
| 20.91690574 | 0.003115265 | 28 | DNMT1 | FALSE | DNMT1 |
| 258.4939506 | 0.003649635 | 55 | JUN | FALSE | JUN |
| 48.12154367 | 0.003030303 | 23 | PTPN1 | FALSE | PTPN1 |
| 1794.17842 | 0.004098361 | 75 | AKT1 | FALSE | AKT1 |
| 254.0135811 | 0.003484321 | 43 | RELA | FALSE | RELA |
| 1689.540999 | 0.004115226 | 75 | TNF | FALSE | TNF |
| 109.6261639 | 0.003289474 | 36 | AR | FALSE | AR |
| 761.4043204 | 0.003745318 | 58 | ESR1 | FALSE | ESR1 |
| 230.4778175 | 0.003623188 | 49 | BCL2 | FALSE | BCL2 |
| 47.66847283 | 0.002469136 | 5 | ACADSB | TRUE | ACADSB |
| 756.2090931 | 0.003636364 | 50 | NR3C1 | FALSE | NR3C1 |
| 374.8912998 | 0.003144654 | 30 | FASN | FALSE | FASN |
| 97.05190064 | 0.002898551 | 14 | ACSL4 | TRUE | ACSL4 |
| 131.6569736 | 0.003174603 | 15 | ACHE | TRUE | ACHE |
| 78.87104284 | 0.003125 | 16 | TYR | TRUE | TYR |
| 308.4234473 | 0.003164557 | 20 | SLC6A3 | FALSE | SLC6A3 |
| 1292.602218 | 0.003891051 | 64 | PTGS2 | FALSE | PTGS2 |
| 127.5298585 | 0.002531646 | 8 | CHRM1 | TRUE | CHRM1 |
| 60.69528276 | 0.002564103 | 17 | SCN4A | TRUE | SCN4A |
| 93.422975 | 0.002754821 | 10 | FADS2 | TRUE | FADS2 |
| 71.83473169 | 0.002398082 | 7 | ADH5 | TRUE | ADH5 |
| 72.6497011 | 0.002747253 | 12 | NR1I3 | TRUE | NR1I3 |
| 65.27893218 | 0.002688172 | 9 | FADS1 | TRUE | FADS1 |
| 90.54393662 | 0.003125 | 28 | NCOA2 | FALSE | NCOA2 |
| 353.8293881 | 0.003030303 | 23 | NR1H4 | FALSE | NR1H4 |
| 91.07251015 | 0.00304878 | 27 | NCOA1 | FALSE | NCOA1 |
| 55.00842345 | 0.003215434 | 23 | NOS2 | FALSE | NOS2 |
| 87.04894995 | 0.003144654 | 28 | RXRA | FALSE | RXRA |
| 117.6806073 | 0.003194888 | 31 | PPARD | FALSE | PPARD |
| 355.1466691 | 0.00330033 | 35 | SREBF1 | FALSE | SREBF1 |
| 604.6820913 | 0.003546099 | 44 | PPARA | FALSE | PPARA |
| 167.0217578 | 0.003333333 | 34 | HSPA5 | FALSE | HSPA5 |
| 53.18175492 | 0.002631579 | 10 | CYP2C8 | TRUE | CYP2C8 |
| 0.439709439 | 0.002409639 | 2 | PPT1 | TRUE | PPT1 |
| 6.191449988 | 0.002272727 | 3 | ADH1C | TRUE | ADH1C |
| 269.8146601 | 0.003021148 | 19 | GSTP1 | FALSE | GSTP1 |
| 50.32820144 | 0.002688172 | 9 | OPRD1 | TRUE | OPRD1 |
| 1.849343909 | 0.002188184 | 3 | GLO1 | TRUE | GLO1 |
| 14.66175508 | 0.002444988 | 4 | CES1 | TRUE | CES1 |
| 4.670554405 | 0.00228833 | 8 | ADRA1B | TRUE | ADRA1B |
| 59.81091788 | 0.002710027 | 12 | SLC6A2 | TRUE | SLC6A2 |
| 257.5537658 | 0.00308642 | 20 | SLC6A4 | FALSE | SLC6A4 |
| 11.57917825 | 0.002392344 | 9 | ADRA2B | TRUE | ADRA2B |
| 52.36343414 | 0.002525253 | 12 | HTR3A | TRUE | HTR3A |
| 125.9022764 | 0.003021148 | 12 | ADRB1 | TRUE | ADRB1 |
| 39.70965309 | 0.002624672 | 11 | ADRA2C | TRUE | ADRA2C |
| 35.72193558 | 0.00257732 | 10 | ADRA1D | TRUE | ADRA1D |
| 614.8446705 | 0.003676471 | 55 | MAPK3 | FALSE | MAPK3 |
| 321.8758191 | 0.002770083 | 15 | ADRA2A | FALSE | ADRA2A |
| 100.1193702 | 0.002873563 | 14 | DRD1 | TRUE | DRD1 |
| 188.389016 | 0.002941176 | 15 | HTR2A | FALSE | HTR2A |
| 0 | 0.002008032 | 2 | CHRM3 | TRUE | CHRM3 |
| 330.6710405 | 0.003125 | 27 | PTGS1 | FALSE | PTGS1 |
| 169.8316414 | 0.002659574 | 18 | SCN8A | TRUE | SCN8A |
| 433.3509298 | 0.00330033 | 28 | OPRM1 | FALSE | OPRM1 |
| 182.3243544 | 0.003225806 | 22 | ADRB2 | FALSE | ADRB2 |
| 192.5912955 | 0.002985075 | 11 | RHO | FALSE | RHO |
| 149.1532149 | 0.003154574 | 18 | CNR1 | TRUE | CNR1 |
| 2.171509018 | 0.002475248 | 4 | LDLR | TRUE | LDLR |
| 291.0316308 | 0.003003003 | 15 | AKR1B1 | FALSE | AKR1B1 |
| 0.638461538 | 0.002183406 | 2 | PMP2 | TRUE | PMP2 |
| 48.2588875 | 0.003021148 | 21 | DPP4 | FALSE | DPP4 |
| 11.81075554 | 0.002666667 | 7 | AKR1C3 | TRUE | AKR1C3 |
| 11.17087063 | 0.002717391 | 7 | HSD11B2 | TRUE | HSD11B2 |
| 15.76273804 | 0.002770083 | 9 | HSD11B1 | TRUE | HSD11B1 |
| 0 | 0.002881844 | 11 | MIF | TRUE | MIF |
| 8.723998157 | 0.00295858 | 16 | CSNK2A1 | TRUE | CSNK2A1 |
| 170.9641733 | 0.002915452 | 15 | PLAT | TRUE | PLAT |
| 1.787687657 | 0.002923977 | 11 | LY96 | TRUE | LY96 |
| 183.0188178 | 0.003067485 | 16 | S100B | TRUE | S100B |
| 33.83691111 | 0.002840909 | 16 | BIRC5 | TRUE | BIRC5 |
| 91.437139 | 0.003225806 | 31 | PGR | FALSE | PGR |
| 45.2321003 | 0.002994012 | 18 | NR3C2 | TRUE | NR3C2 |
| 1.026074997 | 0.002695418 | 4 | PDE3A | TRUE | PDE3A |
| 2.664222577 | 0.00297619 | 17 | NFKB2 | TRUE | NFKB2 |
| 61.82006907 | 0.00310559 | 25 | PLAU | FALSE | PLAU |
| 323.492791 | 0.002932551 | 14 | LCN2 | FALSE | LCN2 |
| 145.1890575 | 0.003508772 | 42 | TLR4 | FALSE | TLR4 |
| 24.24288208 | 0.002849003 | 7 | NPPB | TRUE | NPPB |
| 28.37347373 | 0.002949853 | 15 | ANXA1 | TRUE | ANXA1 |
| 13.63110098 | 0.002923977 | 14 | PPP3CA | TRUE | PPP3CA |
| 39.66762212 | 0.00297619 | 13 | FKBP1A | TRUE | FKBP1A |
| 48.04091018 | 0.003125 | 25 | NFATC1 | FALSE | NFATC1 |
| 7.895363421 | 0.002949853 | 14 | HDAC9 | TRUE | HDAC9 |
| 96.05300459 | 0.00310559 | 20 | PIK3CG | TRUE | PIK3CG |
| 72.79376789 | 0.003154574 | 30 | HDAC2 | FALSE | HDAC2 |
| 1.761140665 | 0.002638522 | 3 | ARF1 | TRUE | ARF1 |
| 14.27017292 | 0.002832861 | 13 | FURIN | TRUE | FURIN |
| 0 | 0.00280112 | 9 | VEGFC | TRUE | VEGFC |
| 667.9710081 | 0.003289474 | 28 | TRPV1 | FALSE | TRPV1 |
| 217.2465315 | 0.003030303 | 23 | CALM1 | FALSE | CALM1 |
| 91.64735627 | 0.002881844 | 14 | LTF | TRUE | LTF |
| 60.62830659 | 0.00297619 | 13 | PLA2G2A | TRUE | PLA2G2A |
| 9.104159883 | 0.002666667 | 9 | TOP2B | TRUE | TOP2B |
| 1.082063938 | 0.002747253 | 7 | SHBG | TRUE | SHBG |
| 3.303510692 | 0.002762431 | 11 | PPP3R1 | TRUE | PPP3R1 |
| 8.937114564 | 0.002717391 | 7 | RORA | TRUE | RORA |
| 2.072301739 | 0.00257732 | 5 | PIM1 | TRUE | PIM1 |
| 7.575127144 | 0.002638522 | 6 | CA2 | TRUE | CA2 |
| 49.26073764 | 0.002544529 | 18 | CACNA2D1 | TRUE | CACNA2D1 |
| 0.205357143 | 0.002192982 | 14 | SCN2B | TRUE | SCN2B |
| 26.90857599 | 0.002531646 | 17 | SCN3A | TRUE | SCN3A |
| 48.01947926 | 0.002610966 | 18 | SCN1A | TRUE | SCN1A |
| 33.46350786 | 0.002583979 | 16 | SCN4B | TRUE | SCN4B |
| 195.5389525 | 0.002808989 | 20 | SCN5A | FALSE | SCN5A |
| 21.60550066 | 0.002512563 | 15 | SCN11A | TRUE | SCN11A |
| 17.04628238 | 0.0025 | 16 | SCN2A | TRUE | SCN2A |
| 53.69001276 | 0.002583979 | 17 | SCN9A | TRUE | SCN9A |
| 21.31626041 | 0.002475248 | 15 | SCN1B | TRUE | SCN1B |
| 53.69001276 | 0.002583979 | 17 | SCN10A | TRUE | SCN10A |
| 0.205357143 | 0.002192982 | 14 | SCN3B | TRUE | SCN3B |
| 9.390529179 | 0.002724796 | 8 | CSNK2B | TRUE | CSNK2B |
| 133.133252 | 0.002873563 | 9 | CTRB1 | TRUE | CTRB1 |
| 106.3449642 | 0.002873563 | 10 | PTGER3 | TRUE | PTGER3 |
| 0 | 0.002207506 | 2 | CNR2 | TRUE | CNR2 |
| 1 | 0.002114165 | 3 | SERPIND1 | TRUE | SERPIND1 |
| 0 | 0.002512563 | 2 | PRSS1 | TRUE | PRSS1 |
| 54.87569521 | 0.002949853 | 19 | CYP27B1 | TRUE | CYP27B1 |
| 31.04476837 | 0.002724796 | 14 | RXRG | TRUE | RXRG |
| 21.7593991 | 0.002702703 | 14 | RXRB | TRUE | RXRB |
| 10.83150379 | 0.002666667 | 8 | PLA2G2D | TRUE | PLA2G2D |
| 0.086956522 | 0.002702703 | 5 | EDNRA | TRUE | EDNRA |
| 2.865129682 | 0.002169197 | 3 | ELOVL4 | TRUE | ELOVL4 |
| 43.63197551 | 0.002673797 | 7 | F7 | TRUE | F7 |
| 27.66520832 | 0.002762431 | 10 | VLDLR | TRUE | VLDLR |
| 0 | 0.002222222 | 2 | LSS | TRUE | LSS |
| 0 | 0.002057613 | 1 | HNF4G | TRUE | HNF4G |
| 38.62307196 | 0.002770083 | 8 | LTA4H | TRUE | LTA4H |
| 0 | 0.002444988 | 3 | MTAP | TRUE | MTAP |
| 0 | 0.002012072 | 1 | PAEP | TRUE | PAEP |
| 14.5903302 | 0.002666667 | 5 | LTB4R | TRUE | LTB4R |

**Table S9 Syndrome-specific pathways in integrated analysis of multi-omics and network pharmacology**

| **Intersection** | **ID Annotation** | **KEGG pathway terms** |
| --- | --- | --- |
| Network pharmacology and metabolomics | hsa04148 | Efferocytosis |
|  | hsa04371 | Apelin signaling pathway |
|  | hsa04020 | Calcium signaling pathway |
|  | hsa04072 | Phospholipase D signaling pathway |
|  | hsa05231 | Choline metabolism in cancer |
|  | hsa05131 | Shigellosis |
|  | hsa05152 | Tuberculosis |
|  | hsa04071 | Sphingolipid signaling pathway |
|  | hsa04080 | Neuroactive ligand-receptor interaction |
| Network pharmacology and microbiomics | hsa05219 | Bladder cancer |
|  | hsa00982 | Drug metabolism - cytochrome P450 |
|  | hsa05133 | Pertussis |
|  | hsa00350 | Tyrosine metabolism |
|  | hsa00592 | alpha-Linolenic acid metabolism |

# Supplementary Figures


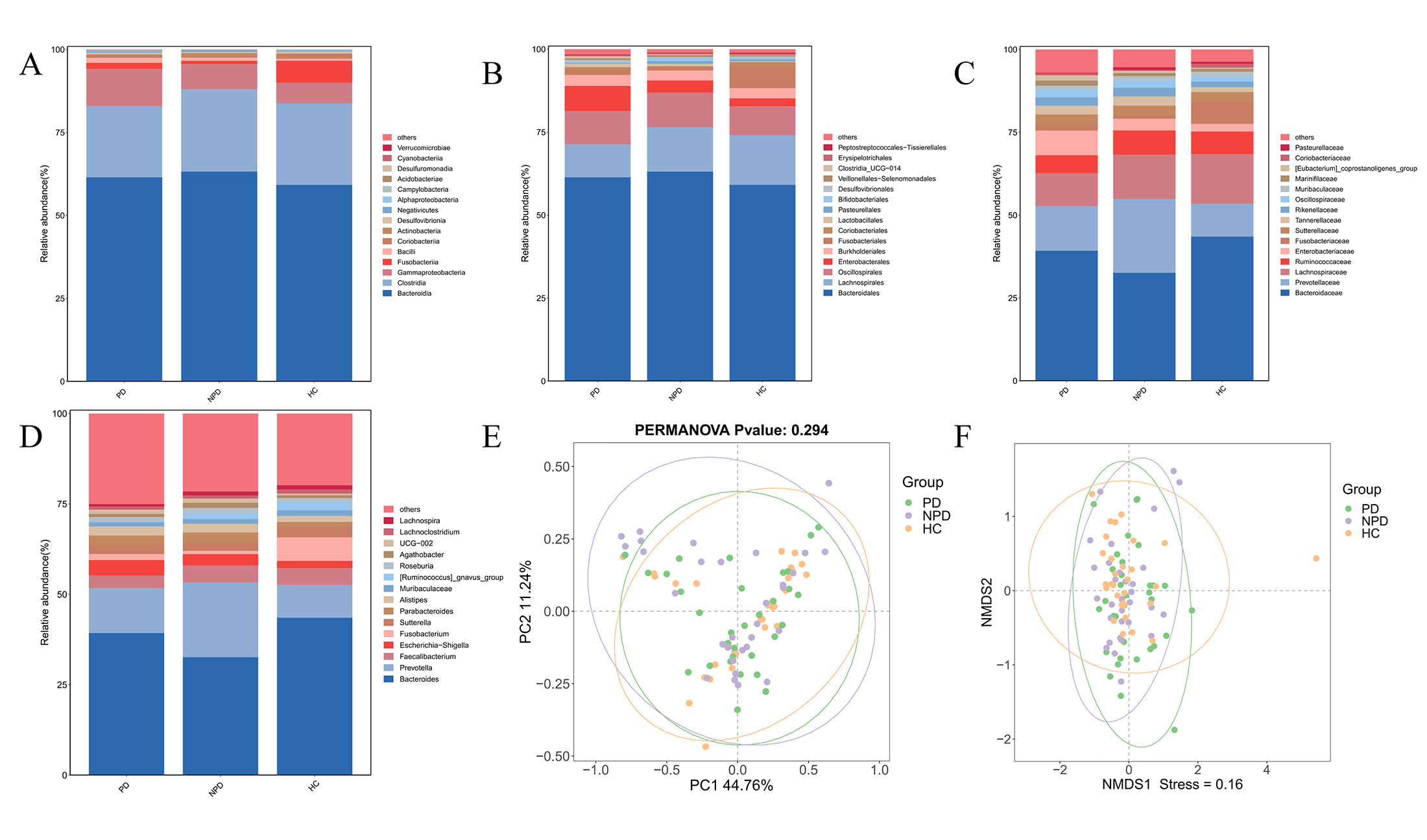


**Supplementary Figure 1.** Microbiomics profiling on IR-CHD patients with PD syndrome, related to Figures 2.

(A) Relative abundance of species at the class level in each group. (B) Relative abundance of species at the order level in each group. (C) Relative abundance of species at the family level in each group. (D) Relative abundance of species at the genus level in each group. (E) PCoA score plots based on weighted UniFrac distances. (F) NMDS score plots. Each point in the graph indicates a sample, and the different point colors indicate the grouping of the sample. The closer the distribution of points, the more similar the sample is.


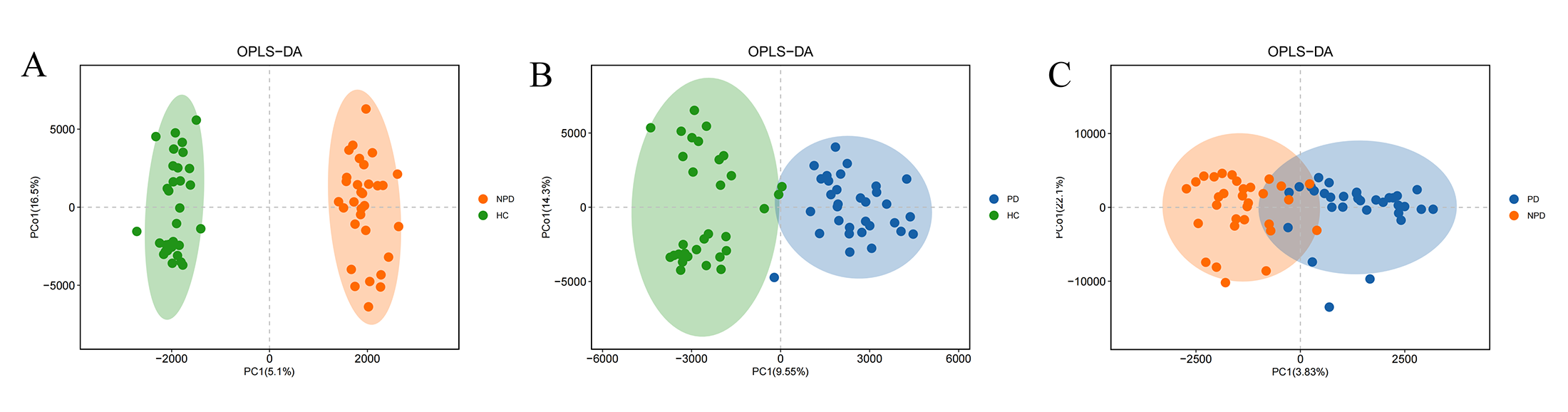


**Supplementary Figure 2.** Metabolomics profiling on IR-CHD patients with PD syndrome, related to Figures 3.

1. PLS-DA plots of the NPD and HC groups. (B) PLS-DA plots of the PD and HC groups. (C) PLS-DA plots of the PD and NPD groups.

**
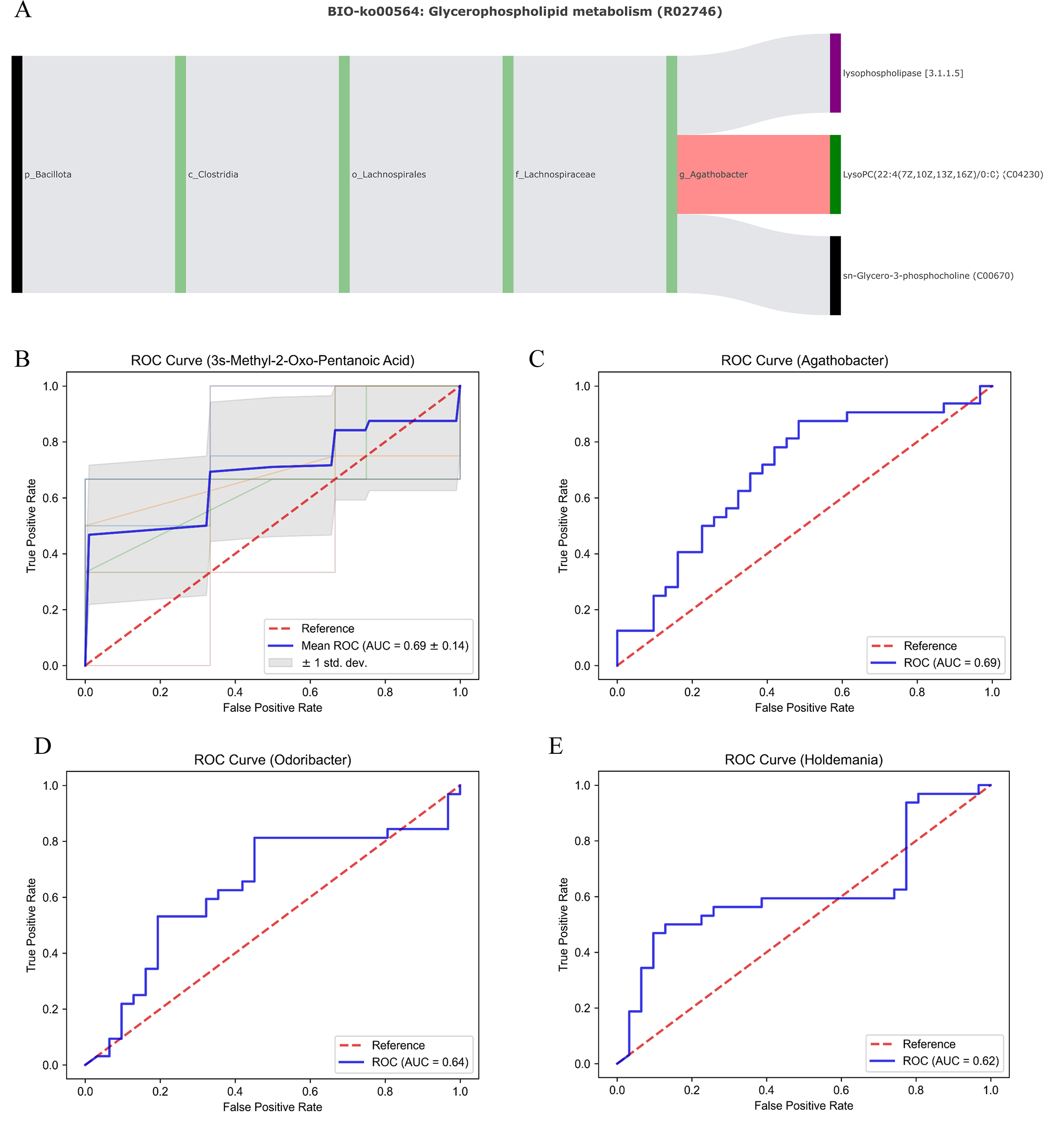
**

**Supplementary Figure 3.** Metabolome-microbiome integrated analysis and ROC diagnosis, related to Figures 5.

1. Sankey diagram of Glycerophospholipid metabolism reaction R02746. (B) The ROC diagnostic analysis of 1 DMs (3-Methyl-2-Oxo-Pentanoic Acid) for PD Syndromes. (C-E) The ROC diagnostic analysis of 3 DMs (*Agathobacter, Odoribacter, Holdemania*) for PD Syndromes.

**
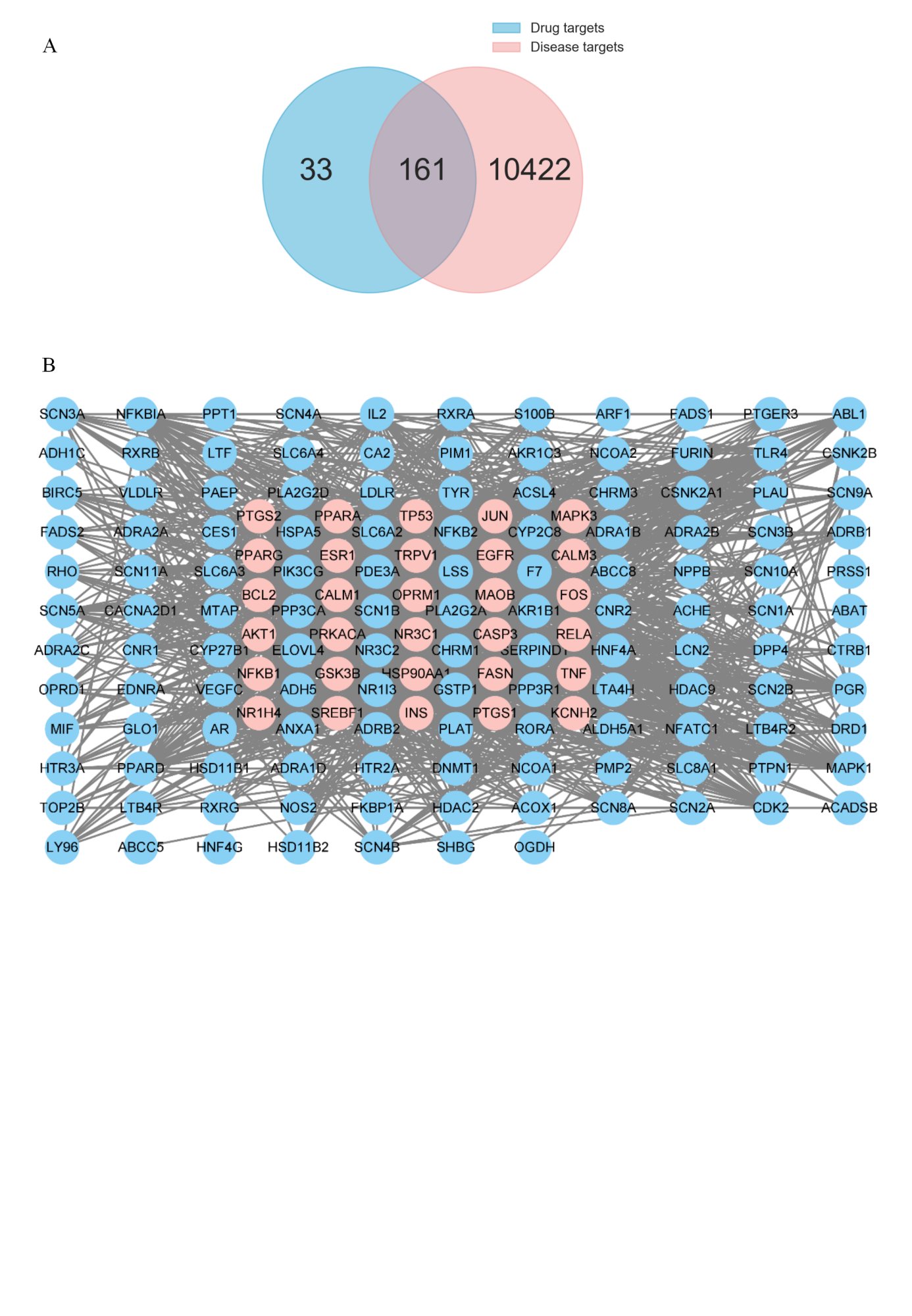
**

**Supplementary Figure 4.** Network pharmacology analysis of TCM formulae used to treat IR-CHD patients with PD syndrome in the clinic, related to Figures 6.

(A) The overlapping signature targets of the PD Syndrome formulae and CHD. (B) The PPI network of the formulae for CHD with PD Syndrome.
